# Molecular underpinnings of metastatic small renal masses

**DOI:** 10.1172/jci.insight.209036

**Published:** 2026-09-22

**Authors:** Payal Kapur, Daria Beshnova, Hua Zhong, Ruby Sharma, Pooja Ghatalia, Angela Yoo, Daniel D. Le, Ratna Mukhopadhyay, Alana Christie, Jeffrey Miyata, Shuanzeng Wei, Rana R. McKay, Dinesh Rakheja, Satwik Rajaram, Robert G. Uzzo, A. Ari Hakimi, Zora Modrusan, James Brugarolas

**Affiliations:** 1 Department of Pathology and; 2 Department of Urology, University of Texas Southwestern Medical Center, Dallas, Texas, USA.; 3 Kidney Cancer Program at Simmons Comprehensive Cancer Center, Dallas, Texas, USA.; 4 Lyda Hill Department of Bioinformatics, University of Texas Southwestern Medical Center, Dallas, Texas, USA.; 5 Department of Surgery, Fox Chase Cancer Center, Temple University School of Medicine, Philadelphia, Pennsylvania, USA.; 6 Department of Surgery, Urology Service, Memorial Sloan Kettering Cancer Center, New York, New York, USA.; 7 Proteomic and Genomic Technologies Department, Genentech Inc., South San Francisco, California, USA.; 8 Department of Internal Medicine (Division of Hematology-Oncology), University of Texas Southwestern Medical Center, Dallas, Texas, USA.; 9 Division of Hematology Oncology, UCSD, San Diego, California, USA.

**Keywords:** Clinical Research, Genetics, Oncology, Molecular pathology

## Abstract

Metastases in renal cell carcinoma (RCC) typically arise from large primary tumors. However, a subset of patients with small renal masses (SRMs; ≤4 cm) can develop metastatic disease. Identifying these tumors is clinically important, as many SRMs are managed with active surveillance, and their study may provide insight into the early acquisition of metastatic competence. It remains unclear whether these tumors acquire distinct metastatic programs or instead show premature activation of the same aggressive programs typically associated with larger tumors. Here, we performed integrated morphological and molecular profiling of a multiinstitutional cohort of metastatic SRMs, including whole-exome sequencing and RNA-Seq, using nonmetastatic primary tumors as controls. Among metastatic, non–clear cell SRMs, we identified *NF2*-altered tumors, *ELOC*-mutated RCC, and an mTOR-driven eosinophilic vacuolated tumor. Metastatic clear cell SRMs were enriched by multi-hit aggressive genotypes, including recurrent losses of chromosomes 8p, 9, and 14q, as well as co-occurring driver alterations (≥2 events), including *BAP1* and mTOR pathway genes. Transcriptomic analyses revealed enrichment of the non-negative matrix factorization 3 (NMF3) subtype from the IMmotion151 trial-based taxonomy, along with metabolic rewiring and reduced cytotoxic immune effector function. Collectively, these findings identify molecular programs associated with metastatic competence in SRMs, highlight the importance of genomic studies of equivocal non-clear cell SRMs, and provide a biological framework for risk stratification in patients often considered for active surveillance.

## Abstract 2

Using integrated pathology, whole-exome sequencing, and RNA-seq of a multi-institutional cohort, this work defines the molecular landscape of metastatic small renal masses and identify features associated with early metastatic competence. #KidneyCancer #CancerGenomics #Pathology

## Introduction

Kidney and renal pelvis cancers account for approximately 80,450 new diagnoses each year in the United States (1). The widespread use of cross-sectional imaging has led to increased detection of incidentally discovered small renal masses (SRMs, ≤4 cm). Management of SRMs has evolved from routine surgery toward a more risk-adapted approach that includes partial nephrectomy, image-guided ablation, and active surveillance, with the latter endorsed by National Comprehensive Cancer Network guidelines for smaller tumors and older or medically frail patients (2). This paradigm is supported in part by the “3-cm rule,” originally developed for hereditary renal cancer syndromes such as von Hippel–Lindau (VHL) disease, in which intervention is generally deferred until the largest renal tumor reaches approximately 3 cm because the risk of metastasis below this threshold is considered low.

Although most SRMs behave indolently, a small (<5%) but clinically relevant subset of SRMs metastasize (3). Unlike breast and lung cancers, which may disseminate despite a small primary tumor, metastatic spread in clear cell renal cell carcinoma (ccRCC), the most common RCC subtype, typically occurs from large primaries, making metastatic SRMs (SRM-Ms) a particularly intriguing clinical and biological exception. However, several uncommon RCC subtypes, including FH-deficient RCC, are recognized to metastasize early despite small primary tumors. Elucidating the mechanisms underlying aggressive behavior in SRMs may therefore improve our understanding of both the rare aggressive histological subtypes and the subset of small ccRCCs that acquire metastatic competence. Their small size may also be advantageous from a biological standpoint, as they may harbor less intratumoral heterogeneity than larger tumors, thereby offering a clearer window into the early events that underlie acquisition of metastatic competence. Yet, dedicated studies of SRM-Ms are scarce, largely because they are uncommon and only a few cases are available at most institutions.

Advances in RCC genomics have refined our understanding and improved risk stratification (4–6). BRCA1-associated gene 1 (*BAP1*) mutations, present in approximately 15% of primary ccRCCs, are consistently associated with high-grade morphology, adverse pathological features, and poor outcome (4, 5, 7, 8). Using an IHC test we developed, we showed that BAP1 loss was prognostic, independently of other features, in a large cohort of low-risk tumors characterized by a SSIGN (stage, size, grade, necrosis) score of 3 or lower (8–10). These findings were validated in a previous institutional analysis of SRMs (11, 12). However, the broader molecular architecture of SRM-M remains undefined. In particular, it is not known whether SRM-Ms harbor distinct drivers, or whether they represent small tumors that have already converged on the same genomic and transcriptional programs that characterize larger, aggressive RCCs.

Here, we address this question through a multiinstitutional integrated morphological and molecular analysis of SRM-Ms, including both clear-cell (SRM-M^ccRCC^) and non–clear cell (SRM-M^non-ccRCC^) histologies. Using centralized pathological review, somatic mutations and copy-number variations from whole-exome sequencing (WES), and gene expression from RNA-Seq, we compared SRM-M primary tumors with pathological stage-matched nonmetastatic primary tumor controls to identify features associated with metastatic behavior. We show that genomic profiling refined the classification of SRM-M^non-ccRCC^ and identified aggressive molecular subsets, including *NF2*-deficient RCCs, *ELOC*-mutated RCC, and an eosinophilic vacuolated tumor (EVT) with metastatic behavior. We further report that SRM-M^ccRCC^ accumulate canonical aggressive events early on, including recurrent chromosome 8p, 9, and 14q losses as well as *BAP1* and mTOR pathway alterations. By RNA-Seq, SRM-M^ccRCC^ exhibited a convergent transcriptional program characterized by metabolic rewiring and attenuated cytotoxic immune function. Together, these findings define early molecular routes to metastatic competence in SRM and provide a foundation for risk stratification of small renal tumors.

## Results

### Clinico-pathological features of SRM-M.

To interrogate the basis of SRM-M, we assembled a multiinstitutional cohort from 3 large-volume centers (see Methods), comprising 86 patients. Key clinicopathological features are summarized in Supplemental Table 1 (supplemental material available online with this article; https://doi.org/10.1172/jci.insight.209036DS1). The median age at nephrectomy was 64 years, and baseline demographics were broadly comparable to previously reported cohorts (12). By design, all primary tumors met SRM size criteria at resection (largest dimension ≤ 4 cm; median 3.0 cm, interquartile range 2.5–3.5 cm). Histologically, tumors were predominantly ccRCC (SRM-M^ccRCC^, *n* = 64). Despite their small size, lymph node involvement at nephrectomy was observed in 9 patients (11%), and a substantial fraction of patients (33/86; 39%) had synchronous metastasis at diagnosis. The remaining patients developed metachronous metastasis, occurring as late as 12 years later (median: 0.98 years). Compared with an institutional SRM-NM cohort (*n* = 1,539 patients), patients with SRM-M were frequently older men and had adverse risk variables such as larger primary tumors; high nuclear grade (G3-4); sarcomatoid features; pT3-4; pN1; and tumor, node, and metastasis (TNM) stage III–IV (all with χ^2^ *P* < 0.001) (Table 1). Compared with SRM-NM, the frequency was similar for ccRCC, lower for papillary and chromophobe RCC, and higher for unclassified and other rare aggressive RCC subtypes (Table 1). Collectively, these clinicopathological features indicate that SRM-M represents a biologically aggressive subset of SRMs, in which metastatic potential is not adequately captured by primary tumor size alone.

### Sample selection for genomic analysis and data integration.

We next established a rigorous, pathology-driven sampling strategy to ensure that downstream genomic analyses were anchored on the most biologically relevant (aggressive) regions of each tumor (most available samples were from primary tumors). All available H&E slides underwent centralized morphological review. For cases not from University of Texas Southwestern Medical Center (UTSW), a single representative slide (requested from the highest-grade area) was reviewed. For UTSW cases, complete clinical slide sets were reviewed, and the highest-grade region was selected for sampling. These areas were then macro-dissected (or subjected to core punching) to extract DNA and RNA. For the UTSW cohort, both primary and metastatic sites were sampled when available to enable within-patient comparisons. This workflow yielded a final analytic cohort of 110 tumor samples (86 from primaries and 24 from metastatic sites) from 86 SRM-M patients (Supplemental Table 2). Out of 81 patients who passed sequencing quality control for DNA, matched normal sample was available for 44 patients. For the remaining 37 patients, nephron-sparing partial nephrectomy limited the availability of adjacent normal kidney tissue (a pooled normal kidney reference from available nonneoplastic samples was used to enable germline-aware variant calling).

To define a clinically relevant comparator for metastasis-specific discovery, we included a control-NM cohort of RCC without metastasis with at least 3 years of follow-up (median 95.2 months). This yielded 19 control-NM^ccRCC^ tumors for WES/copy-number analyses and 18 control-NM^ccRCC^ tumors for RNA-Seq analyses after integration of contemporaneously sequenced controls, with previously published (13) UTSW cases processed using the same sequencing workflow. Because this comparator included a subset of nonmetastatic tumors up to 7 cm, we refer to this group as control-NM^ccRCC^ rather than SRM-NM^ccRCC^. Clinicopathological features of the combined control cohort were balanced and comparable to the SRM-M group (Supplemental Table 1).

### Frequency of driver mutations in SRM-Ms of non–clear cell histology.

**Figure 1:**
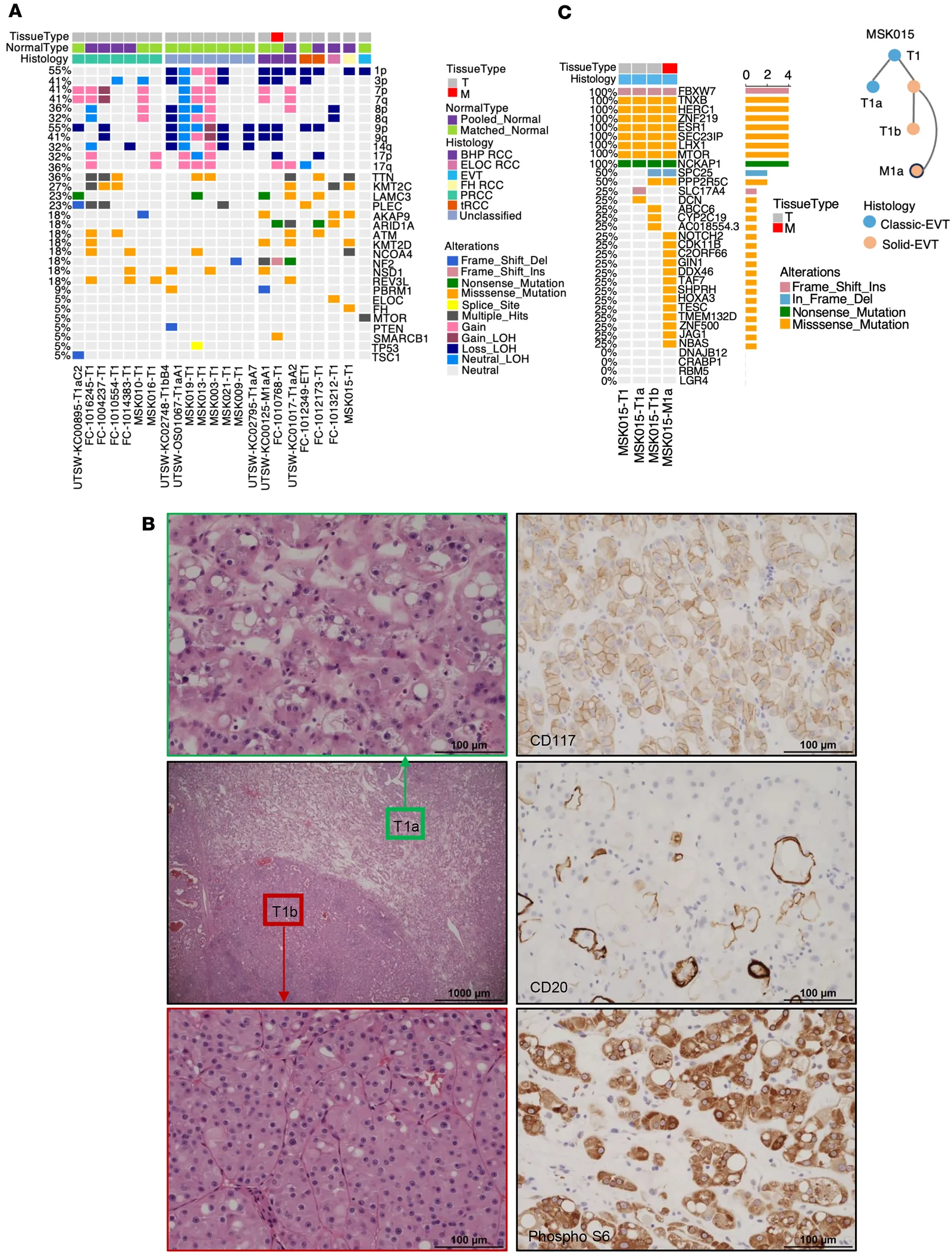
Integrated genomic profiling refines the classification of metastatic non–clear cell SRMs and identifies aggressive molecular subtypes. (**A**) Oncoplot showing recurrent somatic (nonsynonymous) alterations along with variant classification, mutation frequency, and arm-level copy-number changes across SRM-M^non-ccRCC^ cases (*n* = 21), annotated by tissue type (primary or metastasis), normal reference type, and revised histological classification. To avoid duplication, a single sample for MSK015 (T1) is shown. (**B**) Representative H&E images from the eosinophilic vacuolated tumor (EVT-MSK015) show 2 morphologically distinct regions, including a classic EVT component (T1a-higher magnification shown in green box) and a more advanced solid component (T1b-higher magnification shown in red box) (scale bars: 1,000 μm [middle], 100 μm [top and bottom]). Left panels show EVT showing positive membranous CD117, rare focal cytokeratin 20 expression restricted to the classic EVT area, and diffuse phospho-S6 expression (all 200× original magnification). (**C**) Oncoplot along with phylogeny tree from multiregional genomic profiling of the EVT case (4 sampled regions: 2 classic areas, MSK015 T1 and T1a, a solid area, MSK015 T1b, and a lymph node metastasis, MSK015 M1a). Oncoplot shows shared and region-specific alterations. The phylogeny tree suggests that the metastasis arose from the solid area (T1b), which evolved from the classic EVT region (T1/T1a). BHP RCC, biphasic hyalinizing psammomatous renal cell carcinoma; ccRCC, clear cell renal cell carcinoma; Del, deletion; ELOC RCC, ELOC-mutated renal cell carcinoma; EVT, eosinophilic vacuolated tumor; FH RCC, fumarate hydratase–deficient renal cell carcinoma; Ins, insertion; LOH, loss of heterozygosity; M, metastatic tumor; PRCC, papillary renal cell carcinoma; RCC, renal cell carcinoma; T, primary tumor; tRCC, translocation renal cell carcinoma.

We next focused on SRM-Ms of non-ccRCC histology (SRM-M^non-ccRCC^) for which there were 21 tumors with at least 1 sample passing quality control. The original histological classification consisted of 7 papillary RCCs, 1 clear cell papillary RCC, 2 translocation RCCs, and 11 unclassified RCCs. Two cases histologically classified as translocation RCC were genomically confirmed as *TFE3*-rearranged RCC (Figure 1A), a subtype known for metastatic propensity (14, 15). However, genomic profiling analyses identified alterations prompting reclassification of 29% of tumors (6/21). One tumor originally classified as clear cell papillary “RCC” harbored a hotspot ELOC p.Y79 mutation with 8q21 monosomy. *ELOC* (elongin C) is a core component of the VHL ubiquitin ligase complex that regulates HIFα, and though the tumor morphologically resembled ccRCC, given the genomics and stromal enriched nodular growth pattern, the case was reclassified as *ELOC*-mutated RCC, a rare entity that has been reported to metastasize (16). A second tumor originally classified as papillary RCC showed a pathogenic *FH* mutation with chromosome 1p loss. Although the tumor showed papillary architecture with high-grade nuclei, it lacked classic perinucleolar clearing, and FH protein was retained by IHC (likely because the mutation was a point mutation), leading to an erroneous diagnosis. Overall, the integrated molecular profile supported reclassification as *FH*-deficient RCC, a subtype with well-established aggressive behavior (14).

**Figure 2:**
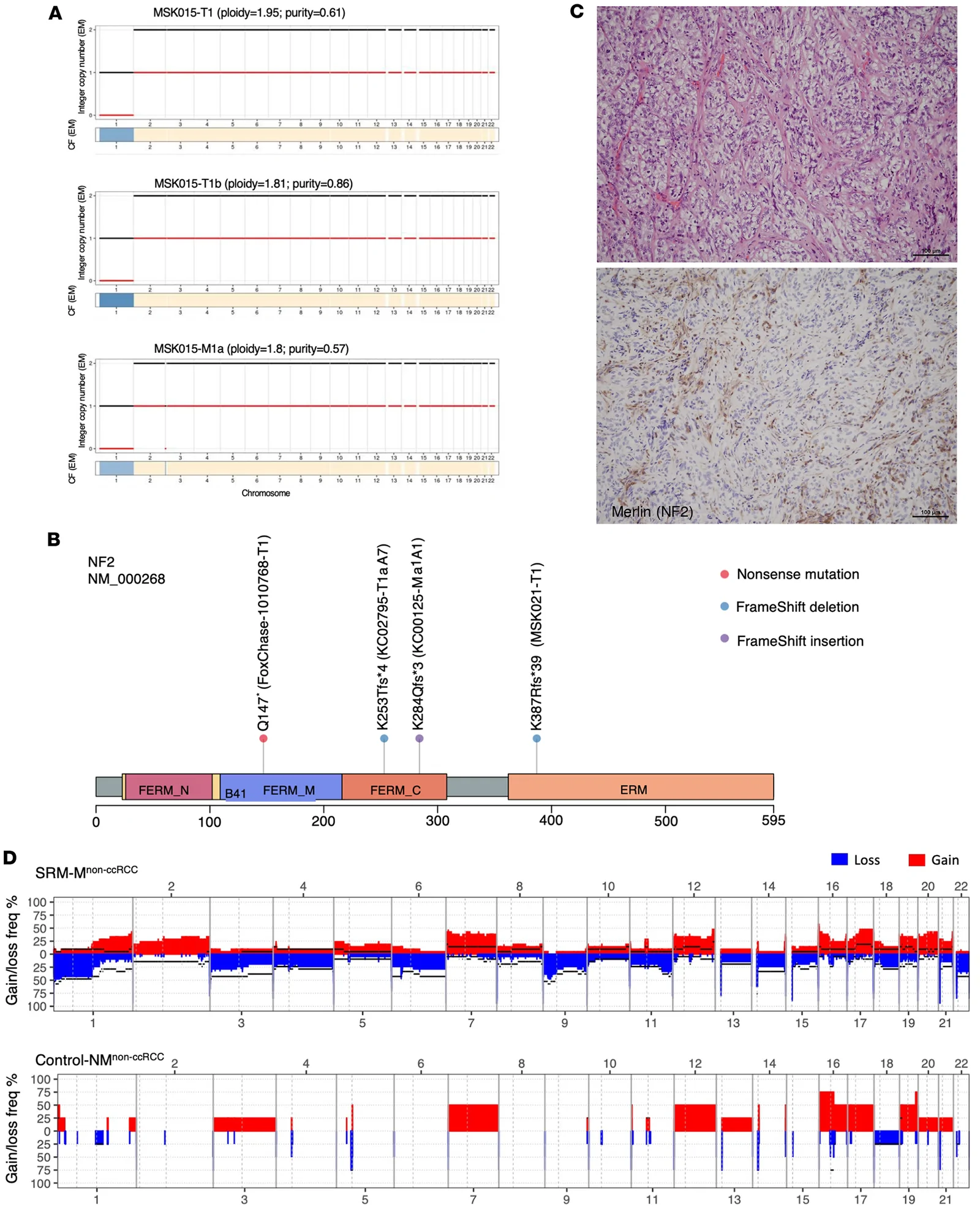
Genomic characterization of metastatic non–clear cell small renal masses reveals NF2 alterations and distinct chromosomal copy-number profiles. (**A**) Representative genome-wide copy-number profile (FACETS) from the classic, solid, and metastatic samples demonstrate similar copy-number alterations. (**B**) Lollipop plot depiction of *NF2* mutations. (**C**) Representative H&E image and corresponding Merlin IHC from an *NF*2-altered unclassified RCC showing infiltrative eosinophilic/tubulo-nested morphology in sclerotic stroma and loss of Merlin expression in tumor cells (scale bar: 100 μm). (**D**) Genome-wide chromosome-arm gain/loss frequency plots comparing SRM-M^non-ccRCC^ and control-NM^non-ccRCC^ tumors (red, gain; blue, loss), illustrating recurrent broad events across the cohort, including canonical papillary RCC alterations (gains of chromosome 7 and 17) and additional arm-level losses associated with metastatic competence including losses of chromosome 9 and 14.

Within the tumors remaining unclassified by morphology, genomic findings also pointed to emerging molecularly defined subtypes. One case harbored a somatic *MTOR* variant (c.7278_7280GCT>AAA; p.Leu2427Lys) with chromosome 1 loss, alterations described in EVT (17, 18). Leucine 2427 is highly conserved, it localizes to the mTOR kinase domain, and mutations of this residue have been associated with mTOR activation (18). This was supported by the strong and diffuse phospho-S6 expression by IHC (Figure 1B). Notably, this tumor exhibited 2 distinct morphological compartments: region 1 showed classic EVT-like features, with nested architecture, hypovascular hyalinized pale stroma, tumor cells with abundant eosinophilic cytoplasm, and prominent cytoplasmic vacuoles (Figure 1B); and region 2 with a more cellular, compact nested/sheet-like growth pattern with diminished vacuolization, accompanied by greater nuclear size variation and focal mitotic activity (2-3/10 high power fields [HPF]). WES of tumor (from region 1, region 2, and metastasis as well as adjacent normal renal parenchyma) supported the EVT classification by confirming the defining somatic alteration and copy-number profile (18, 19) and further supported the metastasis to have originated from region 2, which evolved from region 1 (Figure 1C and Figure 2A). Immunophenotypically, tumor cells were PAX8^+^ (diffuse nuclear), CD117^+^ (membranous), CK20 patchy (restricted to region 1), cathepsin-K^+^, and CK7^–^. These data support the notion that EVT can progress and metastasize.

Finally, among the unclassified RCC group, *NF2* emerged as a recurrent driver. We previously reported the identification of *NF2* mutations in non-ccRCC (20). Subsequently, *NF2* was found to be mutated in RCC by The Cancer Genome Atlas (TCGA) and more recently described as a novel entity by Argani et al. (21–23). Interestingly, 3/10 (30%) unclassified tumors carried pathogenic *NF2* mutations accompanied by 22q loss involving the *NF2* locus, and a fourth tumor had an *NF2* mutation without 22q loss (Figure 1A and Figure 2B). Morphologically, these tumors showed infiltrative borders with tubular and nested architecture in a sclerotic stroma and eosinophilic cytoplasm. In 1 evaluable case, Merlin (NF2) IHC was performed and demonstrated loss in tumor cells with retained staining in the background stromal cells (Figure 2C). Although classic biphasic hyalinizing psammomatous RCC morphology corresponding to an emerging histological entity with *NF2* loss (22) was not observed, these cases should be reclassified as *NF2*-deficient and they expand the morphological spectrum of *NF2*-altered RCC.

At the copy-number level, SRM-M^non-ccRCC^ tumors demonstrated frequent losses, including of chromosome 9 and 14q, consistent with prior reports implicating these events in metastatic competency (24) (Figure 2D). Overall, integrated morphological and genomic profiling in SRM-M^non-ccRCC^ not only clarified diagnostically ambiguous tumors by uncovering defining driver events (e.g., *ELOC*, *FH*, *TFE3*, *NF2*) but also highlighted recurrent copy-number losses (chromosome 9 and 14q) as shared alterations associated with metastatic behavior across diverse non–clear cell histologies.

### Frequency of driver alterations in SRM-Ms of clear cell histology.

Next, we focused on clear cell SRM-M. Analyses were performed on 75 tumors (59 patients) meeting sequencing and variant-calling quality thresholds after removing tumor samples from 5 patients (Supplemental Table 2).

**Figure 3:**
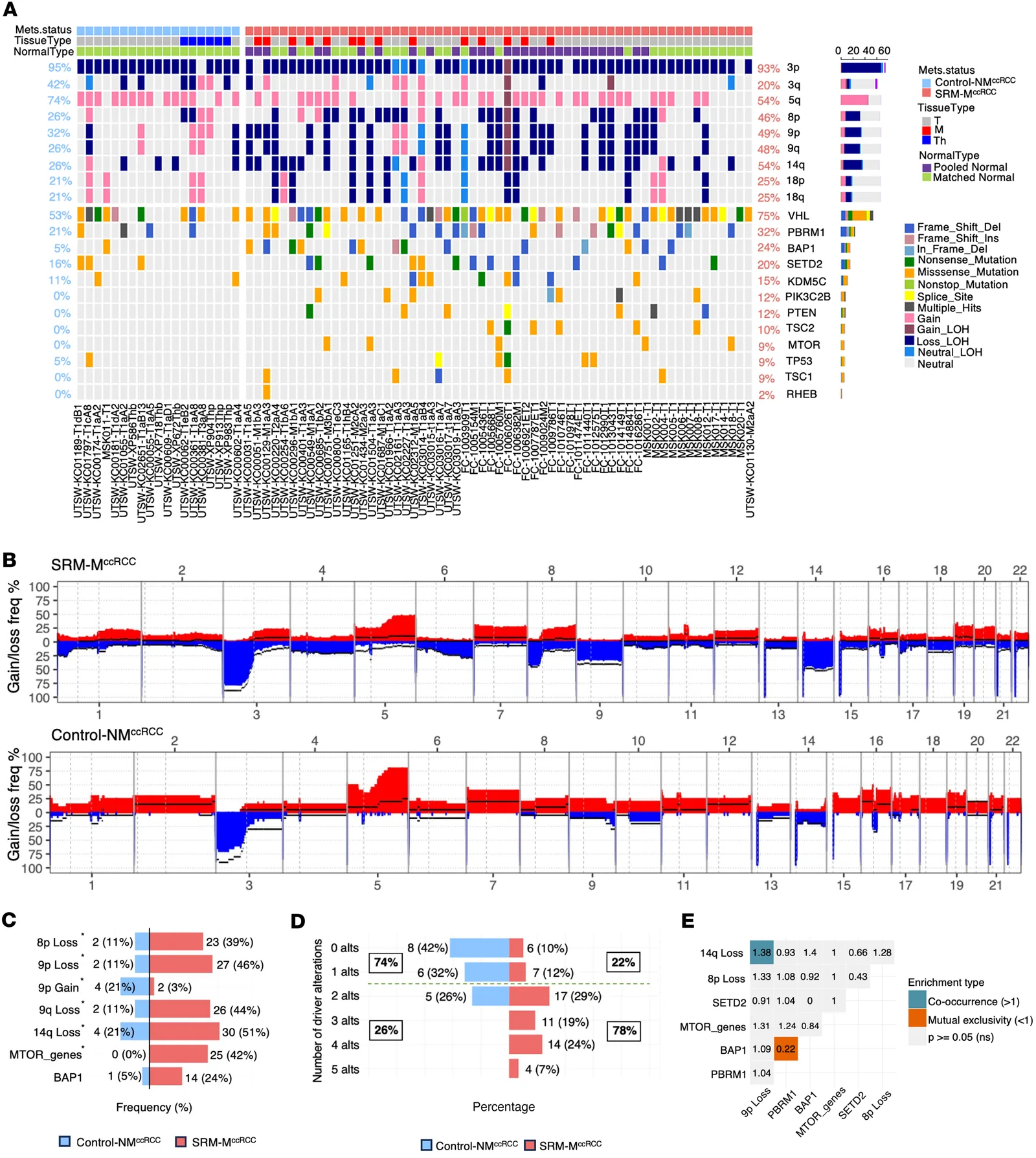
SRM-M^ccRCC^ retains canonical ccRCC drivers but is enriched for multi-hit genomic alterations and metastasis-associated copy-number losses. (**A**) Integrated oncoplot of SRM-M^ccRCC^ (*n* = 59) and control tumors (*n* = 19) showing somatic (nonsynonymous) mutations and arm-level copy-number alterations, annotated by metastasis status, tissue type (tumor vs. thrombus), and normal reference type. For each alteration, the percentage on the left in blue represents its frequency in Control-NM^ccRCC^ tumors, whereas the percentage shown in parentheses on the right in red represents its frequency in SRM-M^ccRCC^ tumors. (**B**) Copy-number gain/loss frequency between SRM-M^ccRCC^ and control tumors. (**C**) Bar plot of selected recurrent alterations shows increased frequencies of 8p/9p/9q/14q loss, mTOR pathway, and *BAP1* alterations in SRM-M^ccRCC^ relative to controls. (**D**) Distribution of the number of metastatic driver alterations per tumor. The boxed percentages summarize the proportions of tumors with 0–1 driver alterations and 2–5 driver alterations, respectively. (**E**) Pairwise co-occurrence/mutual exclusivity matrix of major driver events. LOH, loss of heterozygosity; M, metastasis; T, primary tumor; Th, tumor thrombus.

We first assessed the total mutation burden (TMB; nonsynonymous somatic mutations). Among tumors sequenced with paired normal reference (*n* = 30), we observed 122 putative somatic mutations with a median TMB of 3.4 (range 1.3–8.2). As expected, TMB calling was higher for unmatched tumors using a pooled reference (~5 fold), and to minimize the confounding effect, we focused on established ccRCC driver genes (24–26). Reassuringly, driver event mutation frequencies between matched and unmatched tumors showed no significant differences (Supplemental Table 3). For a subset of patients, multiple samples were available, including paired primary and metastatic specimens in the SRM-M cohort and paired primary and thrombus specimens in the control-NM^ccRCC^ cohort. As shown in Supplemental Figure 1A, we observed that the frequencies were comparable between paired primary and metastasis/thrombus samples, supporting the view that site of sampling did not meaningfully influence the overall driver mutation landscape. Given this concordance, and to avoid overrepresentation of individual patients in cohort-level frequency comparisons, we restricted downstream analyses to 1 sample per patient. When multiple samples were available, we selected the specimen most likely to capture the clinically aggressive subclone, usually the metastasis or renal vein thrombus. We quantified protein-coding alterations in key ccRCC driver genes (Figure 3A and Supplemental Table 4). Analyses were complemented by individual review of each variant using the Integrated Genomics Viewer. Many canonical genes were found to be mutated at frequencies comparable to other studies: *VHL* (75%), *PBRM1* (32%), *SETD2* (20%), and *KDM5C* (15%) and did not differ quantitatively from the reference group (Figure 3A) (25, 26). In contrast, extending our prior observations (12), *BAP1* alterations were more frequent in SRM-M^ccRCC^ than in controls (24% vs. 5%). Although this difference did not reach statistical significance (Fisher’s exact test, *P* = 0.1), the number of sequenced nonmetastatic controls was limited. We therefore extended the comparison using BAP1 IHC in available SRM-MccRCC tumors from the current multiinstitutional cohort and the larger previously characterized institutional cohort of nonmetastatic ccRCC SRMs (12). Loss of BAP1 expression was significantly more frequent in SRM-MccRCC than in nonmetastatic SRMs (13/51 [25.5%] versus 47/685 [6.9%]; Fisher’s exact test, *P* = 8.25 × 10^–5^). These findings support enrichment of BAP1 loss in SRM-M^ccRCC^. Furthermore, we observed a significant enrichment of alterations affecting the mTOR signaling axis (*TSC1*, *TSC2*, *MTOR*, *RHEB*, *PIK3C2B*, and *PTEN*) in SRM-M^ccRCC^ compared with controls (42% vs. 0%, Fisher’s exact test, *P* < 0.001).

Because alterations in *BAP1*, *PBRM1*, and *SETD2* often occur in a background of 3p loss and are frequently associated with loss of protein expression, we used IHC (8) on available material to orthogonally validate the results. We observed loss of nuclear BAP1 expression in 19/21 mutated cases (the 2 discordant cases carried missense variants that may impair function while preserving the protein) (8). Concordant patterns were also observed for PBRM1 and H3K36me3 (a surrogate marker of SETD2 activity), though with lower overall concordance rates (Supplemental Figure 1B). Given the WES results showing enrichment of mTORC1/PI3K-AKT pathway alterations, we evaluated phospho-S6 (Ser240/244) as a surrogate of functional evidence of downstream mTORC1 activation. Phospho-S6 was significantly higher (H-score >40) in SRM-M^ccRCC^ with mTORC1 pathway gene mutations compared with control-NM^ccRCC^ (87% vs. 26%; *P* < 0.01; Supplemental Figure 1B), supporting that recurrent genomic alterations in this pathway are translated into biologically active mTORC1 signaling. Overall, SRM-M^ccRCC^ largely retained the canonical ccRCC driver landscape but showed enrichment for biologically aggressive programs, most notably frequent mTOR-pathway alterations and a trend toward higher *BAP1* mutation, with functional impact supported by orthogonal IHC validation.

### Recurrent somatic copy-number alterations in SRM-Ms of clear cell histology.

We next evaluated the copy-number profile of SRM-M^ccRCC^ (Figure 3B and Supplemental Figure 1C). Consistent with canonical ccRCC biology, the most frequent chromosomal events were 3p loss (93%) and 5q gain (56%). These hallmark alterations occurred at rates comparable to contemporary ccRCC series (13, 25, 27) and did not differ significantly between the SRM-M^ccRCC^ and matched control-NM^ccRCC^ cohorts, suggesting that SRM-M^ccRCC^ retains the foundational ccRCC copy-number backbone. In contrast, SRM-M^ccRCC^ demonstrated a selective enrichment of additional arm-level losses that have been linked to aggressive disease in larger tumors (24, 25). Specifically, whole-chromosome or arm-level losses involving 8p, 9, and 14q were significantly more frequent in SRM-M^ccRCC^ than in controls, with 18p loss showing a strong trend toward significance (*P* = 0.057) (Figure 3, B and C, and Supplemental Figure 1C). These patterns suggest that SRM-Ms acquire a copy-number program typical of invasion and dissemination despite the smallness of the primary tumor.

To localize potential drivers within these broad events, we performed gene-level copy-number analyses (Supplemental Table 5). SRM-M^ccRCC^ showed recurrent focal deletions involving established or putative tumor suppressors and metastasis-associated genes including *CDKN2A/2B* and *MTAP* (9p21.3), *HIF1A* (14q23.2), *TSC1* (9q34), *PSAT1* (9q21.2), *MGAT2* (14q21.3), and *CSMD1* (8p23.2). In contrast, *IRF1* and *SPOCK1* gains on 5q were more frequent in control-NM^ccRCC^, consistent with relative depletion of 5q gain in the metastatic cohort. Recurrent copy-number events showed both regional and cross-arm co-occurrence, including *CDKN2A/CDKN2B/MTAP* loss at 9p21.3, *HIF1A/MGAT2* loss on 14q, and co-occurring events involving 8p and 9 and 9p and 14q (Supplemental Table 6), indicating that these alterations accumulate within the same SRM-M^ccRCC^ tumors. Together, these findings suggest that although SRM-M^ccRCC^ preserved the canonical ccRCC copy number backbone (3p loss, 5q gain), they were enriched for losses of 8p, 9, and 14q, and focal deletions affecting genes linked to cell-cycle regulation (*CDKN2A*/*CDKN2B*), metabolic signaling (*MTAP*, *TSC1*, *PSAT1*), and immune/extracellular matrix regulation (*IRF1*, *CSMD1*, *MGAT2*, *SPOCK1*), consistent with an aggressive copy-number program associated with metastatic competence.

### SRM-M^ccRCC^ accumulate multiple metastatic driver events early.

Metastasis in SRM-M^ccRCC^ spanned a wide clinical interval (0–12 years from nephrectomy; median: 0.98 years) from diagnosis. We asked whether the genomic drivers differed according to the timing of metastatic progression. Specifically, when we compared patients presenting with early (<1 year) metastasis and those who developed late metastases (>3 years), we did not observe statistically significant differences in driver mutation frequencies (Supplemental Figure 1D; global Fisher’s exact test, *P* = 0.13). Similarly, we did not observe statistically significant differences in driver mutation frequencies when comparing samples from patients with synchronous versus metachronous metastasis. Consistent with our prior work (28), however, *BAP1*-altered tumors trended toward earlier metastasis, whereas *PBRM1*-altered tumors showed a trend toward late metastatic progression (>3 years; *P* = 0.087), although these associations were limited by cohort size (Supplemental Table 7).

Next, we asked whether the burden of metastatic driver events differed between SRM-M^ccRCC^ and controls. When we quantified the number of canonical driver alterations per tumor, we found that although individual events were also observed in controls, the presence of 2 or more driver alterations were more common in SRM-M^ccRCC^ (46/59 = 78%) than in the nonmetastatic control cohort (5/19 = 26%; *P* = 0.00008) (Figure 3D). Co-occurrence analysis did not demonstrate enrichment of specific driver combinations, with the notable exception of concurrent 9p and 14q losses (*P* = 0.035) (Figure 3E). Consistent with our previous studies (5, 8), *BAP1* and *PBRM1* alterations remained largely mutually exclusive in this cohort.

We next assessed whether recurrent driver alterations were early/founding events or were acquired and selected during tumor evolution. Compared with primary SRM-M samples, metastatic samples showed a higher fraction of driver-alterations above the clonal cancer cell fraction threshold (cancer cell fraction > 0.5) (Supplemental Figure 1E). This shift from subclonal representation in the primary tumor to clonal representation in the metastasis is consistent with selection and expansion of driver-bearing subclones during metastatic progression.

In summary, SRM-M^ccRCC^ does not show a sharply distinct genotype; instead, it is characterized by an increased early accumulation of multiple metastatic driver events, with frequent multi-hit driver profiles and selective co-occurrence of 9p and 14q losses, features consistent with a previously reported aggressive evolutionary program. What is notable are these developments despite the small primary tumor size.

### Transcriptional analyses reveal site-dominant variation.

We next asked whether metastatic competency in SRM-M^ccRCC^ is accompanied by a characteristic transcriptional state, and whether these programs provide functional support for the genomic alterations identified by WES. RNA was extracted from the same regions used for WES, yielding suitable data from 58 samples (from 49 SRM-M^ccRCC^ patients) and 9 samples (from 9 stage-matched controls) (Supplemental Table 2). To increase the robustness of the control set, we included RNA-Seq data from our prior study (13), which included 9 samples from 9 nonmetastatic patients processed using the same sequencing workflow (18 control-NM^ccRCC^ patient samples in total). After batch correction, normal kidney samples clustered together on uniform manifold approximation and projection (UMAP) as expected, whereas tumor samples showed partial clustering by anatomic site, with primary tumors clustering together, separate from metastatic-site samples (Supplemental Figure 2A). These findings indicated that site contributes substantially to global transcriptional variance. Therefore, to identify transcriptomic features more likely to reflect metastatic competency rather than sampling site, we performed 2 complementary analyses: comparison of primary SRM-M^ccRCC^ tumors with control-NM^ccRCC^, and an orthogonal comparison of SRM-M^ccRCC^ metastatic samples with the same control-NM^ccRCC^.

### Orthogonal differential expression and pathway analyses identify a conserved metabolic and immune-suppression program.

**Figure 4:**
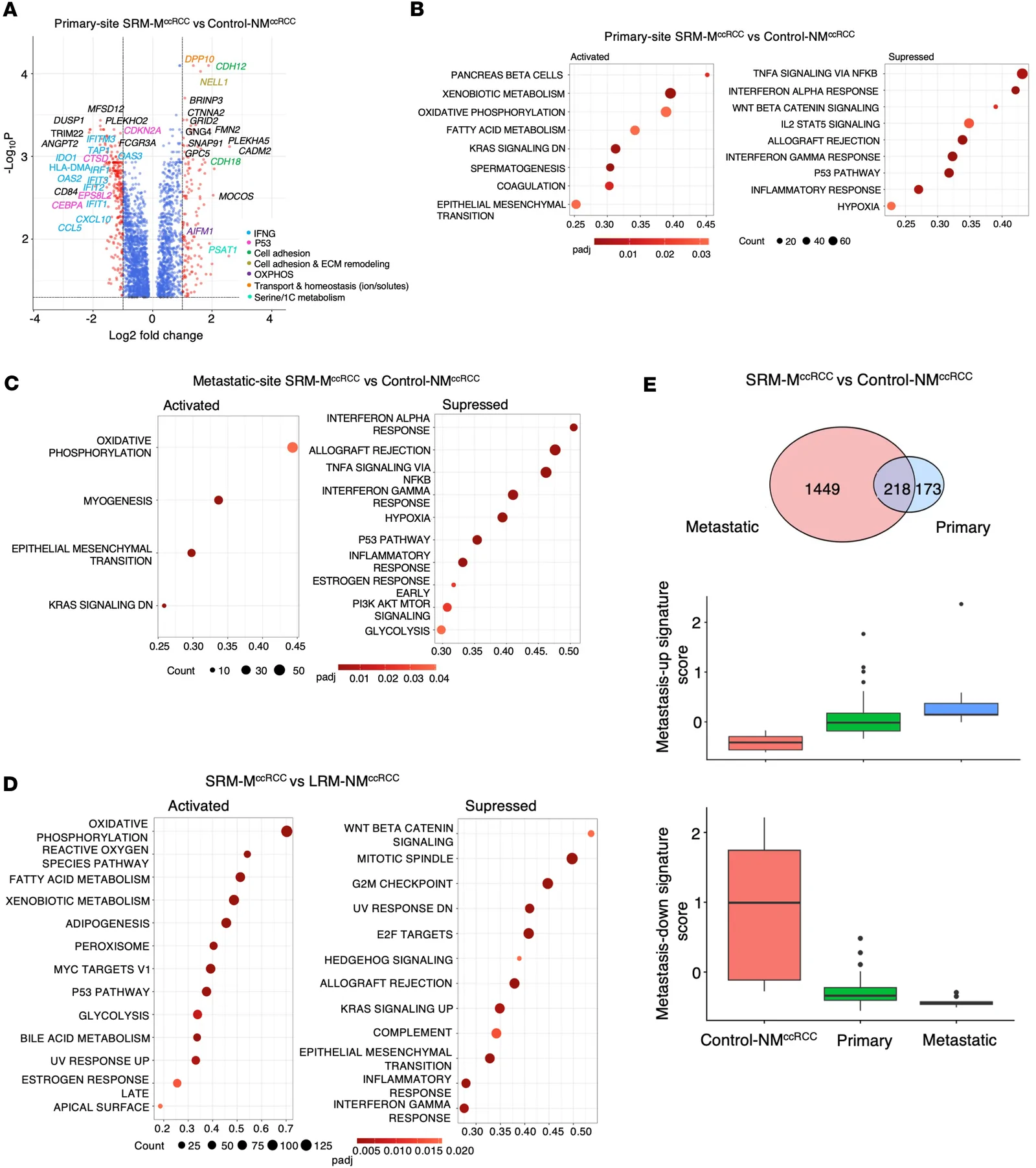
Transcriptional profiling of SRM-M^ccRCC^ reveals metabolic reprogramming and suppression of inflammatory signaling. (**A**) Volcano plot of DEGs between primary samples from SRM-M^ccRCC^ and control-NM^ccRCC^ (393 DEGs total; 173 upregulated, 220 downregulated; ≥2-fold change, FDR < 0.05). (**B**) Hallmark GSEA dot plots of pathways activated and suppressed in primary samples of SRM-M^ccRCC^ versus control-NM^ccRCC^. (**C**) Hallmark GSEA dot plots of pathways activated and suppressed in metastatic samples of SRM-M^ccRCC^ versus control-NM^ccRCC^. (**D**) Hallmark GSEA dot plots of pathways activated and suppressed in SRM-M^ccRCC^ versus LRM-NM^ccRCC^ (larger than 10 cm without metastasis). (**E**) Venn diagram showing 218 overlapping DEGs between primary and metastatic samples from SRM-M^ccRCC^ versus control-NM^ccRCC^. Box plots show the metastasis-associated gene signature score calculated from normalized expression of these 218 upregulated and downregulated gene sets. ECM, extracellular matrix; padj, FDR-adjusted *P* value. GSEA, gene set enrichment analysis; IFN, interferon; LRM, large renal mass; NF-κB, nuclear factor κ B; padj, adjusted *P* value; TNF-α, tumor necrosis factor α.

We first performed differential expression analysis comparing primary tumor samples from SRM-M^ccRCC^ with control-NM^ccRCC^. We identified 393 differentially expressed genes (DEGs) (≥2-fold change, FDR *P* < 0.05), including 173 upregulated and 220 downregulated genes (Figure 4A and Supplemental Table 8). Upregulated genes included those related to oxidative phosphorylation and amino acid metabolism, such as *AIFM1* and *PSAT1*, as well as adhesion-associated genes including *CDH12* and *CDH18*. The downregulated genes mapped predominantly to immune and inflammatory programs (Figure 4A). Consistent with these findings, oxidative phosphorylation–related genes were overexpressed in SRM-M^ccRCC^ compared with control-NM^ccRCC^ (Supplemental Figure 2B).

Hallmark-based enrichment analysis of primary SRM-M^ccRCC^ samples versus control-NM^ccRCC^ showed induction of oxidative phosphorylation and broader metabolic pathways, alongside suppression of TNF-α/NF-κB signaling, IFN response, and other inflammatory programs (Figure 4B). We next asked whether this biology was preserved in an orthogonal comparison using metastatic-site SRM-M^ccRCC^ samples versus control-NM^ccRCC^. We found that the dominant pathway-level signal was maintained in the metastatic-site SRM-M^ccRCC^ versus control comparison (Figure 4C and Supplemental Table 9).

To test whether this transcriptional program was related to metastatic competency, we compared primary SRM-M^ccRCC^ with nonmetastatic large renal masses of ccRCC histology (LRM-NM^ccRCC^; >10 cm, previously published) (13). This analysis showed a similar directional pattern, with relative enrichment of oxidative phosphorylation and other metabolic pathways and suppression of inflammatory and IFN-related programs (Figure 4D). The convergence of these 3 comparisons argues that the observed pathway architecture reflects a reproducible biology associated with metastatic competence in SRM-M^ccRCC^.

Overlap analysis identified 219 genes, of which 218 were concordantly altered (i.e., exhibited the same directional change), across the primary-tumor and metastatic-site comparisons, defining a core metastasis-associated gene signature. Reassuringly, the median expression of this signature showed consistent directional separation from control-NM^ccRCC^ across primary and metastatic-site SRM-M^ccRCC^ samples (Figure 4E).

To further characterize upstream regulatory programs, we performed regulon analysis using decoupleR to identify putative transcription factors (TFs) with differential activity. Eight TFs showed significantly altered activity between SRM-M^ccRCC^ and control-NM^ccRCC^. As shown in Supplemental Figure 2C, the differentiation-associated TF *SIX2* was increased, whereas *FOXP2* was decreased; regulators linked to IFN response (*STAT1*), immune signaling (*STAT5B* and *BMAL1*), and invasion (*ZEB1*) were also reduced. Overall, these TF-level findings are consistent with the broader metabolic and immune-suppressed state observed in the pathway-level analyses.

### NMF subtype assignment supports an angiogenesis-low and metabolically rewired state in SRM-M^ccRCC^.

To define higher-order transcriptional programs, we assigned tumors to previously validated NMF subtypes developed in the IMmotion151 trial (29). SRM-M^ccRCC^ tumors were enriched for NMF3 relative to controls (54.5% vs. 27.8%) (Supplemental Figure 2D). Consistent with this classification, SRM-M^ccRCC^ showed relative enrichment of the complement cascade/omega oxidation signatures and lower angiogenesis-related programs compared with control-NM^ccRCC^ (Supplemental Figure 2E). SRM-M^ccRCC^ NMF3 tumors exhibited multi-hit genomic alterations (>50% harbored ≥3 driver alterations) and included 10/11 *BAP1*-mutated tumors. Together, these data support a transcriptional state characterized by metabolic rewiring and relative suppression of canonical angiogenic programs.

### Transcriptomic data provides functional support for WES-derived alterations and converges on an internally consistent set of metabolic and immune regulatory candidates.

**Figure 5:**
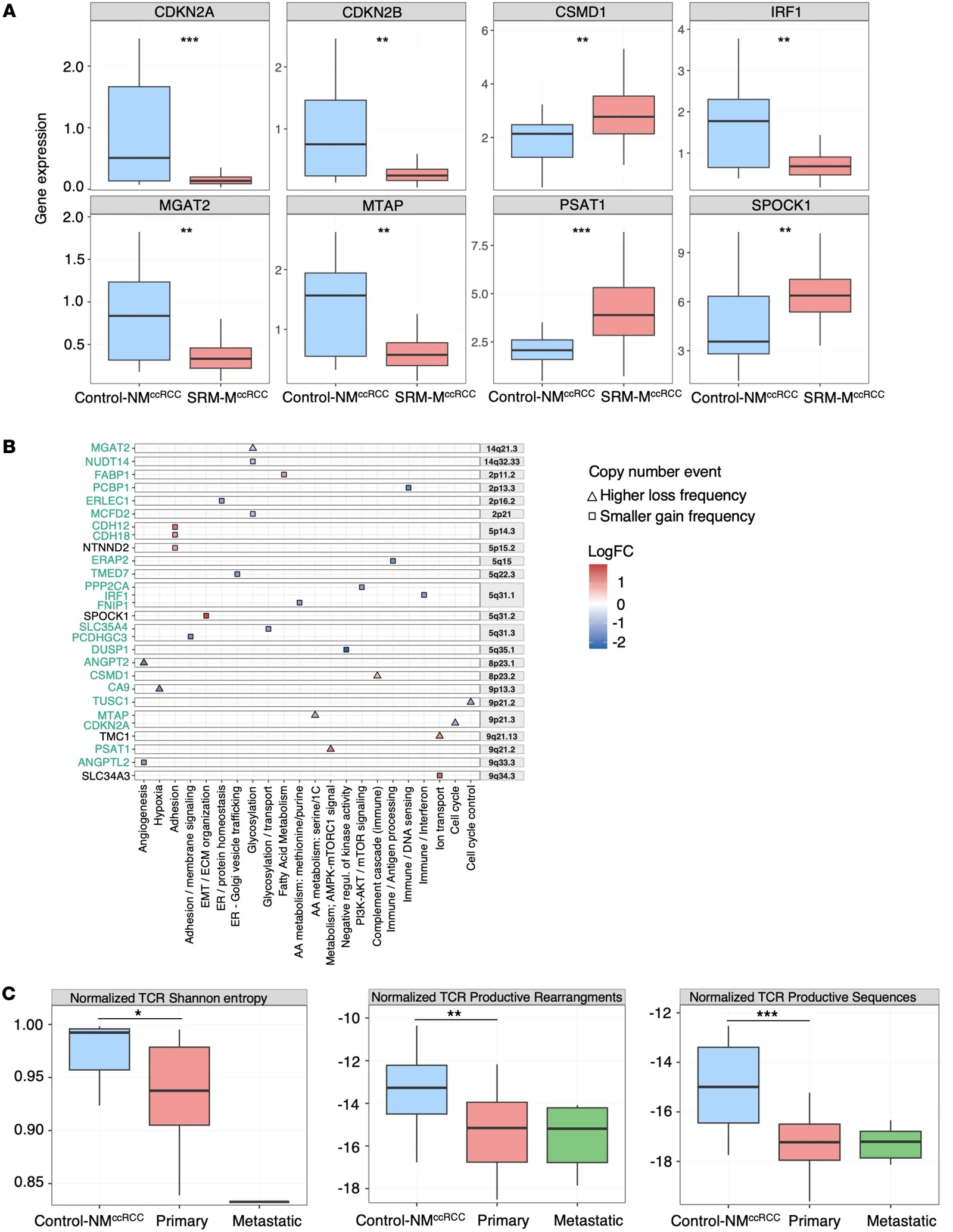
SRM-M^ccRCC^ exhibits immune-related transcriptional and copy-number alterations and reduced T-cell receptor repertoire diversity. (**A**) Boxplots showing expression of selected genes in the DEG analyses between SRM-M^ccRCC^ and control-NM^ccRCC^ with altered copy-number data. (**B**) Integrated WES–RNA pathway plot links relating SCNAs to transcriptional changes. Genes depicted in green represent those that were differentially expressed in samples from both primary and metastasis in SRM-M^ccRCC^ when compared with control-NM^ccRCC^. (**C**) T cell receptor (TCR) repertoire metrics derived from bulk RNA-Seq (including Shannon entropy, productive rearrangements, and productive sequences) were reduced in SRM-M^ccRCC^ (samples from primary and metastasis) relative to control-NM^ccRCC^, supporting decreased T cell diversity/clonal richness and T cell dysfunction. LogFC, log_2_ fold-change; ns, *P* > 0.05; *P < 0.05; **P < 0.01; ***P < 0.001; ****P < 0.0001.

We next integrated gene-level copy number, somatic mutation, and RNA expression data across 49 SRM-M^ccRCC^ and 13 control-NM^ccRCC^ primary tumors with matched WES and RNA to determine whether recurrent genomic alterations were associated with transcriptional change. Several genes showed differential expression by metastatic status, including reduced expression of *CDKN2A*, *MTAP*, *MGAT2*, and *IRF1* and increased expression of *PSAT1*, *CSMD1*, *CDH12*/*CDH18*, and *SPOCK1* in SRM-M^ccRCC^ (Figure 5A). Most notably, *CDKN2A*/*CDKN2B* and *MTAP* remained significantly suppressed in SRM-M^ccRCC^ independent of 9p copy-number effect (*P* < 0.01; Figure 5A), suggesting broader metastatic transcriptional programs.

To prioritize the most biologically relevant candidates, we integrated WES-defined recurrent SCNAs with genes showing differential expression and considered whether these signals were also supported by the orthogonal metastatic-sample analysis (Figure 5B). This integrative approach converged on a smaller internally consistent set of genes and pathways related to metabolism, immune modulation, and aggressive ccRCC biology. Metabolic genes including *FABP1* and *PSAT1* supported the metabolic reprogramming phenotype, whereas *IRF1*, a key regulator of IFN-γ signaling, was significantly downregulated, consistent with suppression of inflammatory immune responses. Overall, the concordance between WES and the 2 orthogonal RNA comparisons strengthens the conclusion that the dominant SRM-M^ccRCC^ transcriptomic signal reflects biologically meaningful programs.

### Immune profiling suggests dysfunction rather than immune cell absence.

Given the recurrent suppression of immune and inflammatory signaling, we next assessed immune cell-type markers and immune-effector programs in greater detail. Expression of CD4, CD8A, and CD8B was comparable to the control, indicating that helper and cytotoxic T cell presence is not broadly different in SRM-M^ccRCC^ (Supplemental Figure 3A). Likewise, canonical checkpoint/exhaustion-associated transcripts (*CTLA4*, *LAG3*, *PDCD1* [PD-1], *CD274* [PD-L1], *TIGIT*) were not enriched (Supplemental Figure 3B), arguing against a dominant classical exhaustion program. However, cytotoxic effector activity appeared attenuated: granzyme B (*GZMB*) was significantly reduced (*P* < 0.05), and IFN-γ showed a nonsignificant downward trend in SRM-M^ccRCC^ (Supplemental Figure 3C). Immune deconvolution (quanTIseq) further suggested a shift toward innate immune remodeling, with increased neutrophil signatures and reduced NK cell abundance in SRM-M^ccRCC^ tumors, although these compositional estimates should be interpreted cautiously (Supplemental Figure 3D). Together, these findings support a model of immune dysfunction rather than simple immune-cell absence in SRM-M^ccRCC^.

To further interrogate adaptive immune dynamics, we applied TRUST4 (30), which infers T and B cell receptor repertoires from bulk RNA-Seq (31–33). Because bulk RNA-Seq–based repertoire inference can be influenced by variation in viable tumor fraction, immune infiltrate abundance, RNA quality, and sequencing depth, we interpreted TRUST4-derived metrics as relative measures of immune receptor recovery from bulk RNA-Seq rather than absolute estimates of lymphocyte abundance. Across SRM-M^ccRCC^ tumors, we identified 251 unique TCR clonotypes in primary tumors and 21 in matched metastatic tumors, along with 3,083 unique BCR clonotypes in primary tumors and 172 in metastatic tumors overall. Median TCR clonotype counts were 2 per sample in both primary and metastatic tumors, whereas median BCR clonotype counts were 20 and 6 per sample, respectively. In contrast, controls exhibited higher repertoire richness, with 1,013 unique TCR clonotypes (median 2.5 per sample) and 48,202 unique BCR clonotypes (median 36.5 per sample) (Supplemental Table 10). Thus, compared with controls, SRM-M^ccRCC^ tumors (both primary and metastasis) showed lower TCR transcript abundance, reduced TCR repertoire richness/diversity (Shannon entropy), and lower fractions of productive rearrangements (Figure 5C). Similar patterns were observed for normalized productive BCR rearrangements (*P* = 0.0012). These findings are consistent with diminished adaptive immune activity and complement the reduced cytotoxic effector signaling observed at the gene-expression level. Taken together, the immune analyses indicate that SRM-M^ccRCC^ tumors are not immune deserts but rather harbor immune cells with attenuated cytotoxic and repertoire features.

## Discussion

We investigated whether SRM-Ms are biologically distinct or represent early activation of aggressive programs typically seen in larger tumors. Using a multiinstitutional cohort with integrated morphological review, WES, copy-number profiling, and RNA-Seq, we identified genomic and transcriptional features associated with metastatic competence. Collectively, our findings support that SRM-Ms constitute a biologically aggressive subset that acquires key metastatic programs early in tumor evolution.

This study builds on our prior single-institution analysis, in which we identified BAP1 loss as an independent predictor of metastatic progression in SRM-M^ccRCC^ that complemented TNM stage for risk stratification (10, 12). In the current multiinstitutional, molecularly profiled cohort, we extend these observations by showing that BAP1 alterations remain enriched in SRM-M^ccRCC^, although the comparison did not reach statistical significance, likely reflecting the limited number of sequenced nonmetastatic controls. When the analysis was extended using BAP1 IHC to the larger previously characterized institutional nonmetastatic SRM cohort, BAP1 loss was significantly more frequent in metastatic tumors (25.5% versus 6.9%). The consistent enrichment of BAP1-altered tumors across cohorts and the strong concordance between genomic alteration and BAP1 protein loss support BAP1 as an early determinant of aggressive biology and a clinically tractable biomarker for risk assessment in SRM (5, 8). More broadly, the genomic landscape of SRM-M^ccRCC^ remained consistent with that of advanced ccRCC, including the expected spectrum of canonical driver alterations, mutually exclusive *BAP1* and *PBRM1* alterations, and recurrent 9p/14q loss (24, 27).

A major strength of this study is its multiinstitutional inclusion of both clear cell and non–clear cell SRM-M (34–36). Our findings illustrate the value of integrating histopathology with genomic profiling in diagnostically challenging or high-risk SRMs. Several tumors were reclassified into molecularly defined entities, including *FH*-deficient RCC and *ELOC*-mutated RCC, underscoring the value of genomic profiling. We also identified an MTOR-mutated eosinophilic renal neoplasm with EVT-like morphology that exhibited morphological progression and metastatic behavior, further supporting the emerging recognition that EVT is not invariably indolent (37, 38). In addition, recurrent *NF2* alterations (3/10; 30%), frequently accompanied by 22q loss, defined a subset of aggressive unclassified non–clear cell SRMs with features extending beyond those described in the emerging entity of biphasic hyalinizing psammomatous RCC, suggesting that the morphological spectrum of NF2-driven tumors is broader than currently appreciated (22, 23, 39). Given the aggressive biology, a classification based primarily on *NF2* loss may be better than reliance on morphological features, which, as described, are absent in a subset of cases. Finally, our identification of a metastatic *ELOC*-mutated RCC arising as an SRM reinforces that even tumors considered relatively indolent may nevertheless acquire metastatic potential (16, 40). Taken together, these findings support incorporating genomic profiling into the evaluation of diagnostically ambiguous or clinically aggressive non–clear cell SRMs.

Within the clear cell subset, metastatic SRM-M^ccRCC^ appeared genomically advanced despite their small size. Although the frequencies of canonical proximal drivers (*VHL*, *PBRM1*, *SETD2*, *KDM5C*) were generally similar to published ccRCC cohorts and our nonmetastatic controls (8, 21, 25), metastatic SRM-M^ccRCC^ were enriched for *BAP1* alterations and, most strikingly, alterations in the mTOR signaling axis (including *TSC1*, *TSC2*, *MTOR*, *RHEB*, *PIK3C2B*, *PTEN*) and multiple (≥2) concurrent metastatic driver events. Together, these findings suggest that metastatic competency arises through early accumulation of canonical high-risk alterations rather than acquisition of SRM-specific genomic events, resulting in tumors that are biologically more similar to advanced ccRCC than their small size would suggest (13, 27). This interpretation is further supported by copy-number analyses showing enrichment for losses involving chromosomes 8p, 9, and 14q (with 18p loss also trending toward significance), alterations previously associated with metastatic progression and poor clinical outcome (24, 27). At the gene level, the recurrent loss of loci including *CDKN2A*/*CDKN2B*/*MTAP* (9p21), *TSC1* (9q34), and *HIF1A* (14q23) is notable, as these genes converge on cell-cycle control, metabolism, and stress-response pathways that plausibly support metastatic dissemination (41). In addition, we found that the transcription factor (TF) *SIX2* was overexpressed in SRM-M^ccRCC^. *SIX2* is reactivated in clear cell tumors, where it can increase migration, and has been shown to promote stemness and metastasis in other cancer types (42–44).

The transcriptomic analyses provide functional support for the genomic findings. Comparison with stage-matched nonmetastatic controls identified a distinct transcriptional program. SRM-M^ccRCC^ were enriched for the NMF3 subtype (IMmotion151 framework), which in our cohort was associated with *BAP1*-alterations, multi-driver events, reduced angiogenic/proliferative signaling, and increased complement/omega oxidation signatures (29). Together, these findings suggest that metastatic SRM-M^ccRCC^ adopt a transcriptional state that differs from the classic hypervascular phenotype of small localized ccRCC but instead reflects a state characterized by metabolic and inflammatory remodeling.

Consistent with this, differential expression and pathway analyses demonstrated induction of oxidative phosphorylation, lipid metabolism, and amino-acid metabolism, along with suppression of innate immune and inflammatory response programs. These transcriptional changes parallel the enrichment of mTORC1/PI3K-AKT pathway alterations and phospho-S6 IHC expression, supporting functional activation of mTOR signaling. Integrated DNA-RNA analyses further identified coordinated dysregulation of metabolic (*PSAT1* and *FABP1*) and immune-related (*IRF1* and *CSMD1*) genes, suggesting that metastatic SRM-M^ccRCC^ are characterized by a coordinated metabolic reprogramming rather than isolated genomic alterations. Notably, despite preserved CD4/CD8 transcript levels and absence of canonical T cell exhaustion markers (e.g., *PDCD1*, *CTLA4*, *LAG3*, *TIGIT*), SRM-M^ccRCC^ exhibited reduced *GZMB* expression and lower TCR/BCR repertoire diversity, consistent with impaired cytotoxic immune function rather than immune exclusion. Together, these findings suggest that early metastatic competency is accompanied by impaired immune function.

This study has several limitations, including its retrospective design and the rarity of SRM-Ms limited sample size, particularly among non–clear cell tumors. In addition, sampling and histological review varied across institutions. Not all cases had matched normal tissue, and RNA analyses incorporated both primary and metastatic tumors, potentially introducing biological and technical heterogeneity despite batch correction. Nevertheless, these findings provide several important insights into the biology of SRM-Ms. Our data suggest that SRM-M^ccRCC^ are characterized by early accumulation of multiple canonical metastatic driver events, including recurrent 9p/14q loss as well as BAP1 and mTOR pathway alterations. It broadens the biological understanding of metastatic non–clear cell SRM, including *NF2*-altered tumors, *ELOC*-mutated RCC, and an EVT with metastatic behavior. Collectively, these findings support a model in which a subset of SRMs acquires metastatic competency early through convergent genomic and transcriptional programs. This report provides a framework for future biomarker-driven risk stratification strategies supporting rational therapeutic interventions in patients with SRMs.

## Methods

### Sex as a biological variable.

This study included both men and women with RCC. Patient sex was recorded as part of the clinical data and was not used as an inclusion or exclusion criterion.

### Patient selection.

We performed a multicenter retrospective study of patients with SRM-Ms treated prior to 2022 at 3 large academic centers including Memorial Sloan Kettering Cancer Center (MSKCC), Fox Chase Cancer Center, and UT Southwestern Medical Center. Patients were considered to have SRM-M if they had primary RCCs 4 cm or smaller with synchronous or metachronous metastasis. Patients with multiple RCCs were not included. No other restrictive inclusion criteria were applied other than tissue availability. The control-NM group included patients without metastasis with more than 3 years of follow-up, and a primary tumor size of 7 cm or smaller.

### Data collection.

Associated data on patient demographics, tumor characteristics, and outcomes were collected using a deidentified data collection template with strict definitions for data collection to minimize interobserver variation. The associated clinicopathological and follow-up information were shared and integrated. The primary outcome assessed was the presence of distant lymph node and/or organ metastasis. The clinical data of the patients included in the UTSW cohort has been previously published (12).

### Sample nomenclature.

Sample identifiers indicate the contributing institution, followed by a deidentified patient number, sample type, and optional block identifier. For the sample type, T denotes primary tumor, M denotes metastasis, and ThP and ThD denote the proximal and distal portions of a tumor thrombus, respectively. For cases with multiregion sampling, an alphabetic character following the sample-type designation identifies the sampled region. The final optional component of the identifier denotes the corresponding tissue-block ID when available.

### Next generation sequencing.

For all patients, representative H&E-stained slide(s) from each sample (primary and/or metastases) were centrally reviewed to assess the region for genomic analyses by evaluating tumor grade, morphological intratumor heterogeneity (ITH), and the extent of nontumor tissue on the slide by a genitourinary pathologist. When sections lacked prominent nontumor tissue and extensive ITH, 30 μm thick scrolls were obtained. When the section included nontumor areas or had prominent ITH, the highest-tumor grade areas and matched normal kidney were marked, and a punch was obtained from the corresponding FFPE block. To enable integrative genomic analyses, both DNA and RNA were simultaneously extracted from the same sample as described previously (45), and sequencing was performed as previously described (20) with whole exome libraries sequenced on average 75 million reads per sample (average read depth >100×) for exome sequencing using 75 bp paired-end on a HiSeqX Plus (Illumina) and RNA-Seq libraries sequenced on average 50 million reads per sample for RNA-Seq using 50 bp single-end on HiSeqX Plus. Our cohort of SRM-M included 36 patients (30 ccRCC and 6 non-ccRCC) from UTSW, 30 patients (22 ccRCC and 8 non-ccRCC) from Fox Chase Cancer Center, and 20 patients (12 ccRCC and 8 non-ccRCC) from MSKCC. For 10 patients, multiple primary and/or metastasis areas were sampled. We concurrently sequenced 18 pT stage-matched SRM-NM with more than 3 years of follow-up. To expand our control group, we integrated data from 9 ccRCC patients from a previously published UTSW cohort (all with available RNA-Seq and 6 with WES data) who had pT3 stage and size 7 cm or smaller (control-NM^ccRCC^) that did not metastasize with minimum 3-year follow-up (13). From the same study (13), in a separate analysis we performed differential gene expression analyses with14 patient samples with renal masses larger than 10 cm (LRM-NM^ccRCC^).

### Somatic mutation calling from WES.

WES reads from FASTQ files were aligned to the human reference genome GRCh38 (hg38) using BWA algorithm (v0.7.15-r1140, RRID:SCR_010910) set to default parameters (46). Picard (v2.18, RRID:SCR_006525) was used to mark PCR duplicates. GATK toolkit (v4.1.4.1, RRID:SCR_001876) (47–49) was used to perform base quality score recalibration and local realignment around insertions and deletions (indels). For tumor samples with paired adjacent normal samples sequenced, Strelka2 (v1.0.15, RRID:SCR_005109) (50) was used to call somatic variants and small-scale indels. For other tumor samples without paired normal tissue, Mutect2 (51) (RRID:SCR_026692) was used to call somatic variations with public GATK panel-of-normal (PON). Although pooled-normal approaches are widely used in the literature (52), we found that GATK PON yielded substantially higher mutation calls (586 putative mutations; median TMB 16.3, range 10.7–54) for these tumor samples without paired normal tissue. Further, we found that benchmarking this approach in cases with available paired normal samples showed systematic inflation of somatic calls when pooled normal was substituted for patient-matched germline reference (data not shown). Annovar (RRID:SCR_012821) was used to annotate somatic mutations and indels (53). Intronic, splice regions, 3′ flanking regions, untranslated regions, and intergenic and silent mutations were filtered out. A variant allele frequency of 5% or greater in tumor samples was required to call a somatic variant. Ensembl Variant Effect Predictor (release 99, RRID:SCR_007931) (54) was used to assign putative functional consequences. Variants were classified according to the American College of Medical Genetics and Genomics (RRID:SCR_005769) 2015 Guidelines. cBioPortal (www.cbioportal.org, RRID:SCR_014555), COSMIC (https://cancer.sanger.ac.uk/cosmic, RRID:SCR_002260), and ClinVar (www.ncbi.nlm.nih.gov/clinvar/, RRID:SCR_006169) were used to annotate cancer relevance and clinical potential for detected variants. Genes depicted in the oncoplots were individually inspected using Integrated Genomics Viewer (v 2.13.1; Broad Institute, MIT Harvard, RRID:SCR_011793) (55).

### Somatic copy-number calling from WES.

Somatic allelic copy-number variation analyses were carried out on paired tumor and normal WES samples using FACETS (v0.6.2, RRID:SCR_026264) (56) and FACETS suite (v2.0.8) R packages; for cases without a matched normal sample, a pooled normal sample with reads obtained from normal samples in the same cohort was substituted. Based on human common variation sites from dbSNP (57) (RRID:SCR_002338), FACETS evaluated read coverage of chromosomal segments and estimated purity, ploidy, and total and allelic integer copy-number for tumor samples. The FACETS suite provided a wrapping function to the FACETS algorithm enabling a 2-pass run to calculate overall copy number and sample purity first and then detect more focal events with increased sensitivity. Two passes were performed with critical values set to 1,000 and 500, respectively, to tune the coarseness of chromosome segmentation. Plots of copy-number log ratio, B-allele logOR, and integer copy number were produced by the FACETS suite. By matching gene locations, gene-level integer copy numbers were inherited from the segment integer copy number from the second pass run. Chromosome arm-level gain or loss was called when more than 50% of the chromosome arm had copy-number gain or loss.

### Gene expression from RNA-Seq.

RNA-Seq raw data were analyzed using HTSeqGenie (58) from Bioconductor (RRID:SCR_006442). Reads with low nucleotide qualities (70% of bases with quality ≤23) or matched to rRNA were removed prior to alignment. Adapter sequences were similarly removed. The remaining reads were aligned to the human reference genome (vGRCh38.p10) using GSNAP (59, 60) (2013-10-10-v2, RRID:SCR_005483), with the following parameters: “-M 2 -n 10 -B 2 -i 1 -N 1 -w 200,000 -E 1 --pairmax-rna=200,000 --clip-overlap” and maximum of 2 mismatches per 75 base sequence. Transcripts were annotated based on the Gencode (61) (RRID:SCR_014966) human genes database (v27). Gene expression levels were quantified by the number of reads mapped unambiguously to the exons of each gene using FeatureCounts (62) (RRID:SCR_012919). Low-count genes were filtered out using the R package edgeR (v3.38.4, RRID:SCR_012802) (63) by requiring at least 10 read counts in at least 2 tumor samples. Normalization was conducted among all high-quality tumor RNA-Seq samples (3 low-quality samples were excluded) in this cohort using the TMM (64) algorithm from edgeR. DEG analysis was performed by edgeR to identify DEGs (absolute log fold-change ≥ 1 and FDR ≤ 0.05) between 44 primary-site SRM-M^ccRCC^ and 18 control-NM^ccRCC^ and separately with 14 LRM-NM^ccRCC^ patients. We performed preranked GSEA with fgsea (v1.32.4, RRID:SCR_020938). The DEG analysis was also performed between 9 metastatic-site SRM-M^ccRCC^ and 18 control-NM^ccRCC^ patients. The log fold-changes represent model-based estimates of expression differences between groups. In all comparisons, the control group (control-NM^ccRCC^ or LRM-NM^ccRCC^) was used as the reference. Therefore, positive log fold-change values indicate higher expression in SRM-M^ccRCC^, whereas negative values indicate higher expression in the control group. Hallmark and C5 GO Biological Process gene sets (65, 66) were obtained from MSigDB (via msigdbr, v25.1.1, RRID:SCR_016863), and significance was defined at FDR *q* ≤ 0.05 (Benjamini–Hochberg). Regulon analysis was performed using Bioconductor package decoupleR (67) (v2.12.0, RRID:SCR_027127) to extract the activity of TFs from expression data. Differential TF activity was tested on quantified activity levels by 2-tailed *t* test between groups for each TF. Bioconductor packages edgeR and sva (v3.44.0, RRID:SCR_002155) (68) were used to perform normalization and batch effect minimization (using ComBat, ref. 69, RRID:SCR_010974) on count data. UMAP (70) (RRID:SCR_018217) plots were generated by R package umapr (v0.0.0.9001) (71) using all filtered genes with default parameters. We reconstructed the random forest model trained on transcripts per million (TPM) expression of the top 10% most variable genes from the IMmotion151 trial cohort (29) using R package Caret (72) with repeated 10-fold cross-validation and smote method to adjust data imbalance. The NMF cluster of SRM tumor samples was predicted by the model given the TPM expression of same genes. For co-occurrence analysis, we constructed a binary alteration matrix (samples × features). For each feature pair (i, j), we computed the observed co-alteration proportion:
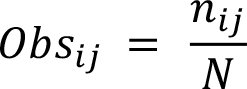


where *n*_i,j_ denotes the number of samples altered in both features *i* and *j*, and *N* is the total number of samples. The expected co-alteration proportion under independence was defined as:
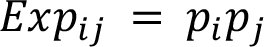


where 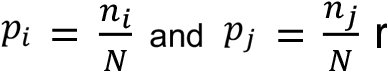 represent the marginal alteration frequencies of features *i* and *j*, respectively. Co-occurrence was summarized using the enrichment ratio:
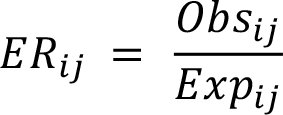


(values >1 indicate enrichment; <1 depletion). Statistical significance of co-occurrence was assessed by Fisher’s exact test. Immune infiltration was quantified by quanTIseq algorithm implemented in TIMER2.0 (73) (RRID:SCR_018737) on the bulk RNA-Seq cohort, yielding relative abundances for B, CD4^+^ T, CD8^+^ T, neutrophil, macrophage, and dendritic cells.

An angiogenesis score was calculated using genes corresponding to the angiogenic cluster identified by NMF clustering. Specifically, expression values for *VEGFA*, *KDR*, *ESM1*, *PECAM1*, *ANGPTL4*, and *CD34* were standardized (*z* score) across samples, and the score was defined as the mean *z*-scored expression of these genes. An OXPHOS score was calculated using leading-edge genes derived from GSEA of SRM-M^ccRCC^ versus control-NM^ccRCC^. Expression values for these genes were similarly standardized (*z* score) across samples, and the OXPHOS score was defined as the mean *z*-scored expression across the leading-edge gene set.

To evaluate whether recurrent genomic alterations were associated with transcriptional changes, we performed a gene-level concordance analysis integrating RNA expression, somatic copy-number alteration, and somatic mutation data. Analyses were restricted to samples with matched expression, gene copy number, and assigned metastasis status, resulting in 49 SRM-M^ccRCC^ and 13 control-NM^ccRCC^ tumor samples. For each candidate gene, normalized log_2_-transformed expression values were extracted, and gene-level copy-number calls were encoded as loss of heterozygosity (LOH), neutral, or gain. Binary indicators were generated for copy-number loss/LOH, copy-number gain, and somatic mutation status. Mutation status was coded as mutated if any qualifying somatic mutation was detected in that gene and not mutated otherwise. For the *MTOR* pathway composite, *MTOR*, *TSC1*, *TSC2*, and *RHEB* were also evaluated as a combined feature, with mutation status coded as altered if any of these genes was mutated.

Mutation clonality was estimated using a cancer cell fraction (CCF) framework integrating somatic mutation allele counts with FACETS-derived purity and allele-specific copy-number estimates. For each mutation, alternate and reference read counts were obtained from the mutation calls, and the number of mutation-bearing tumor cells was estimated after adjustment for the corresponding major allele copy number. Tumor cellularity was derived from FACETS purity for samples with matched normal controls; when FACETS purity was unavailable or pooled normal samples were used, tumor cellularity was inferred from VHL mutation allele fractions. Mutation-level CCF was calculated as the ratio of estimated mutation-bearing tumor cells to total tumor cells, and mutations with CCF of 0.5 or greater were classified as clonal, whereas mutations with CCF less than 0.5 were classified as subclonal.

Arm-level copy-number clonality was estimated from FACETS segment output for recurrent chromosome-arm events. LOH segments were defined by zero minor allele copy number (lcn = 0). For each chromosome arm of interest, an arm-level cellular fraction was calculated as the length-weighted mean of FACETS segment cellular fractions across LOH segments overlapping that arm. Arm-level LOH events with cellular fraction of 0.5 or greater were classified as clonal, and those with cellular fraction less than 0.5 were classified as subclonal.

### T cell immune repertoire.

TCR repertoires were inferred from bulk RNA-Seq data using TRUST4 (30) (RRID:SCR_026162). Productive CDR3 amino acid sequences across TCR loci (*TRA*, *TRB*, *TRG*, and *TRD*) were extracted and analyzed. For each sample, the total number of productive rearrangements and unique productive clonotypes was quantified and normalized to sequencing depth. TCR diversity was assessed using normalized Shannon entropy based on clonotype frequencies, with normalization to the total number of unique clonotypes per sample to account for differences in repertoire size. Samples with insufficient clonotype counts were excluded from diversity analyses. BCR repertoires were similarly inferred from bulk RNA-Seq by extracting productive immunoglobulin rearrangements across *IGH*, *IGK*, and *IGL* loci. Productive BCR clonotypes were summarized as total productive rearrangements and unique productive sequences, with normalization to sequencing depth where applicable.

### IHC.

To confirm the WES analyses, supportive IHC staining was performed on a representative slide when available for BAP1, PBRM1, H3K36me3 (a surrogate for SETD2 and henceforth referred to as SETD2), and phospho-S6 ribosomal protein using an Autostainer Link 48 (Dako, RRID:SCR_026889), as previously described (8, 9, 74). Primary antibodies were obtained from Bethyl Laboratories (PBRM1, 1:4,000 dilution, A301-591A, RRID:AB_1078808), Santa Cruz Biotechnology (BAP1, 1:700 dilution, sc-28383, RRID:AB_626723), Active Motif (H3K36me3, 1:4,500 dilution, 61021, RRID:AB_2614986), and Cell Signaling Technology (phospho-S6, 1:300 dilution, 5364S, RRID:AB_10694233). Appropriate positive and negative controls were incorporated in each IHC run. All stains were scored by a pathologist without knowledge of the associated genomic or clinical information. For BAP1, PBRM1, and H3K36me3, tumors were characterized as negative (loss of the protein/marker) when they lacked nuclear staining in the presence of positive staining in intratumoral lymphocytes and stromal cells that served as internal positive controls. Cases lacking staining of intratumoral lymphocytes and stromal cells were considered uninterpretable and were removed from analysis. Phospho-S6 staining was quantified using the H-score (75). Briefly, phospho-S6 staining intensity was scored as 0 (negative), 1 (weak), 2 (moderate), or 3 (strong) and was multiplied by the percentage of tumor cells expressing the stain.

### Statistics.

All features in the outcome data were summarized with event frequencies, percentages, or median and IQR, as appropriate. Time to metastasis was calculated from the date of nephrectomy to the date of first documented metastasis, last follow-up, or death, whichever was first. Unless indicated, *P* values are 2-sided without adjusting for multiple comparisons. *P* < 0.05 was used to determine statistical significance. Statistical analyses and visualization were conducted using R (version 4.2.0, RRID:SCR_001905).

To assess whether tumors processed with a pooled normal sample were comparable to those with a patient-matched normal sample, we computed the absolute standardized mean difference (76) for the driver alterations.

### Study approval.

This study was conducted with approval by the UTSW IRB (STU 022015-015). Before sending tissue and deidentified data, IRB approval or exemption was obtained at each institution. Informed consent was not required per the IRB because data collected and shared were deidentified.

### Data availability.

Values for all data points in the graphs are available in the Supporting Data Values file.

## Author contributions

PK designed the research studies, acquired and analyzed the data, wrote the manuscript, and secured funding for the project. DB, HZ, RS, DDL, and JM conducted the experiments. DB, PG, AY, RM, and SR analyzed the data. AC, SW, RRM, RGU, and AAH acquired the data. DR provided reagents. ZM oversaw genomic data acquisition. RGU, AAH, JB, DB, and PK reviewed and edited the manuscript. JB assisted with design, interpretation, manuscript writing, and project development. PK conceptualizing the ides, led the entire project, wrote the initial draft, and provided the funding to support the project including DB’s salary. DB conducted part of the NGS analyses under PK’s guidance.

## Conflict of interest

The authors have declared that no conflict of interest exists.

## Funding support

This work is the result of NIH funding, in whole or in part, and is subject to the NIH Public Access Policy. Through acceptance of this federal funding, the NIH has been given a right to make the work publicly available in PubMed Central.

- NIH Clinical Center (SPORE grant P50CA196516) (to PK, JB, AC, JM)
- DOD Peer Reviewed Cancer Research Program (grant KC220107) (to PK, DB, HZ)

## Supplementary Material

Supplemental data

Supplemental tables 1-10

Supporting data values

## Figures and Tables

**Table 1:**
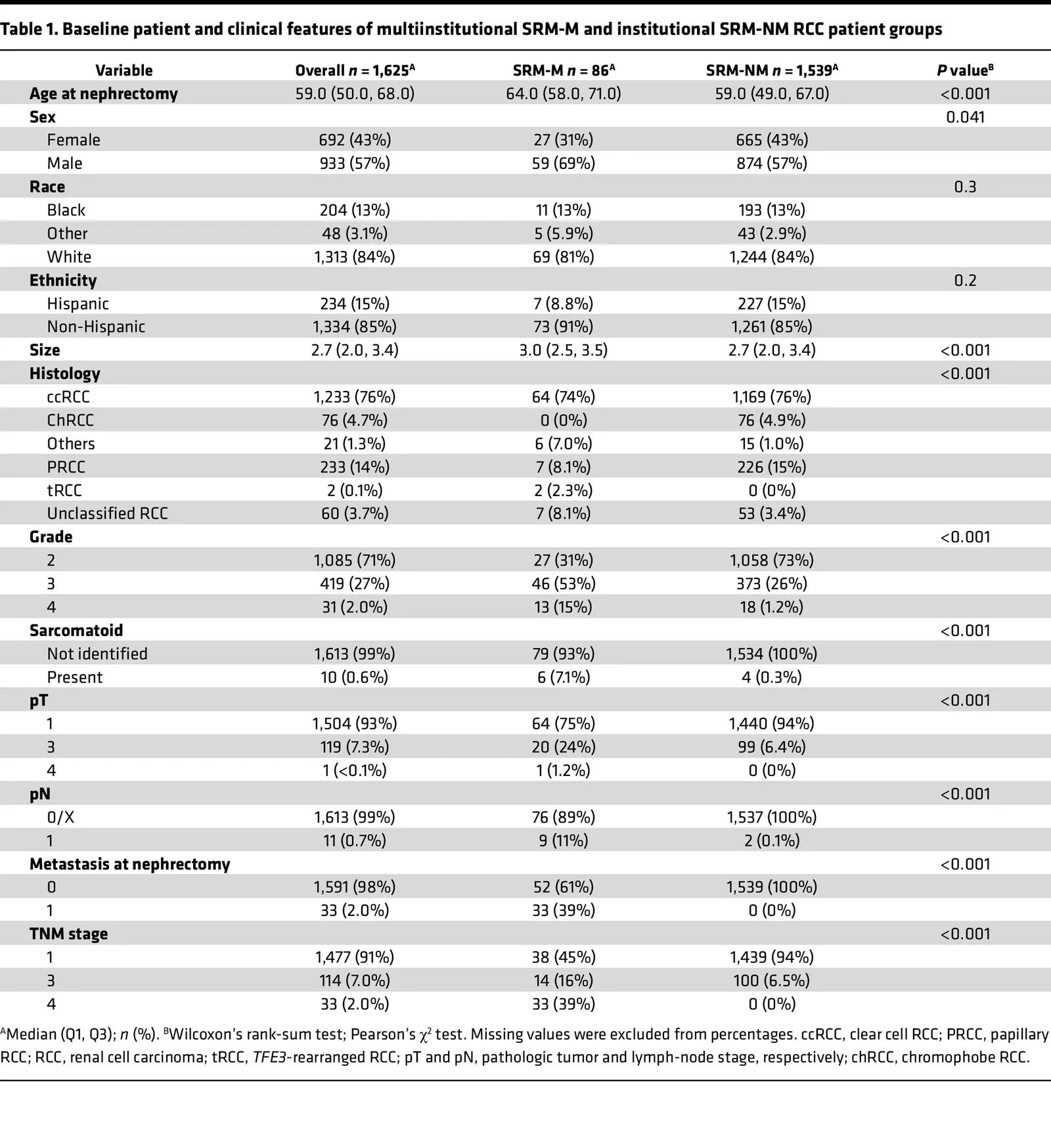
Baseline patient and clinical features of multiinstitutional SRM-M and institutional SRM-NM RCC patient groups

## Version 1 09/22/2026

Electronic publication
