# Supplementary material for: Molecular underpinnings of metastatic small renal masses
Source: JCI Insight. 2026 Sep 22;11(18):e209036. doi: 10.1172/jci.insight.209036 (PMC13596717; doi:10.1172/jci.insight.209036)

Supplemental Figures 1-3

Figure Legends

Supplemental Figure 1. Sample-level genomic features and orthogonal protein validation across the SRM-M^{ccRCC} cohort. (**A)** Expanded oncoplot showing sample-level mutation and copy-number calls across SRM-M^{ccRCC} and control specimens, including multi-region/paired samples and metadata annotations (tissue type, and germline reference type). **(B)** Heatmap comparing mutation status and immunohistochemical readouts for BAP1, PBRM1, and SETD2 (using H3K36me3 as a surrogate), illustrating high concordance between genomic alteration and protein loss, with missense variants annotated separately. Phospho-S6 IHC H-score comparison shows increased mTORC1 pathway activation in SRM-M^{ccRCC}, supporting functional consequences of recurrent alterations in the mTOR signaling axis. **(C)** Chromosome-arm heatmap (gain/neutral/loss) across the cohort stratified by metastasis status, showing recurrent losses in metastatic SRM-M^{ccRCC}, including 8p, 9, and 14q. **(D)** Driver-group distribution (e.g., *BAP1*-driven, *PBRM1*-driven, ≥3 drivers, and others) in patients presenting with early metastasis (<1 years) versus those developing late metastasis (>3 years) post nephrectomy, illustrating similar driver-type composition across metastatic timing groups. (E) Violin and box plots showing cancer cell fraction (CCF) estimates for driver alterations in primary tumors (top) and available metastatic samples (bottom). Each point represents an alteration in an individual sample. The dashed horizontal line indicates the threshold used to classify alterations as clonal versus subclonal. Stacked bar plots showing the proportion of samples in which each recurrent alteration was classified as clonal or subclonal in primary tumors and metastatic samples. Alt, alterations; CCF, cancer cell fraction; IHC, immunohistochemistry; LOH, loss of heterozygosity; M, metastasis; mTORC1, mechanistic target of rapamycin complex 1; T, primary tumor; Th, tumor thrombus, WT, wild type.

Supplemental Figure 2. RNA-seq integration, non-negative matrix factorization (NMF) subtype assignment, and mTORC1 pathway validation in SRM-M^{ccRCC}. **(A)** Uniform manifold approximation and projection (UMAP) of batch-corrected RNA-seq data shows non-neoplastic kidney samples clustering separately, while tumor samples cluster primarily by anatomic site (primary vs metastasis) rather than by metastatic competency alone. **(B)** Boxplots of oxidative phosphorylation related gene expression in SRM-M^{ccRCC} compared with controls. **(C)** Activity level heatmap of differentially activated transcription factors across SRM-M^{ccRCC} and control samples.
(D) NMF-based expression classification (IMmotion151 framework) with heatmap and subtype distribution pie charts shows enrichment of NMF3 in SRM-M^{ccRCC} (including complement/omega oxidation signatures) relative to controls. **(E)** Boxplots of angiogenesis related gene expression in SRM-M^{ccRCC} compared with controls. *, $p<0.05$; **, $p<0.01$. AMPK, AMP-activated protein kinase; FAO, fatty acid oxidation; NMF, non-negative matrix factorization; OXPHOS, oxidative phosphorylation; RNA-seq, RNA sequencing; snoRNA, small nucleolar RNA; UMAP, uniform manifold approximation and projection.

Supplemental Figure 3. Immune checkpoint, cytotoxic, and immune-cell composition analyses support functional immune suppression in SRM-M^{ccRCC}. **(A)** Boxplots of CD4, CD8A (gene that encodes alpha chain of CD8 receptor), and CD8B (gene that encodes beta chain of CD8 receptor) expression show no significant differences between SRM-M^{ccRCC} and control tumors, indicating preserved immune-cell presence. **(B)** Boxplots of immune checkpoint/exhaustion-associated transcripts (*CTLA4*, *HAVCR2*, *LAG3*, *PDCD1*, *TIGIT*) show no significant enrichment of a canonical T-cell exhaustion program in SRM-M^{ccRCC} versus controls. **(C)** Boxplots of cytotoxic lymphocyte-associated genes (*CST7*, *GNLY*, *GZMA*, *GZMB*, *IFNG*, *NKG7*, *PRF1*) show selective reduction of *GZMB* (and a trend for *IFNG*) in SRM-M^{ccRCC}, suggesting an impaired effector function. **(D)** Immune deconvolution (quanTIseq) summary plot shows differential immune-cell composition in SRM-M^{ccRCC} compared with controls, including relative enrichment of neutrophil/M2-like macrophage signatures and reduced NK/M1-like macrophage signals. NK, natural killer; ns, $p>0.05$; *, $p<0.05$; **, $p<0.01$; ***, $p<0.001$; ****, $p<0.0001$. FC, fold change; M1, classically activated macrophage; M2, alternatively activated macrophage; NK, natural killer; ns, not significant.

Supplemental Figure 1

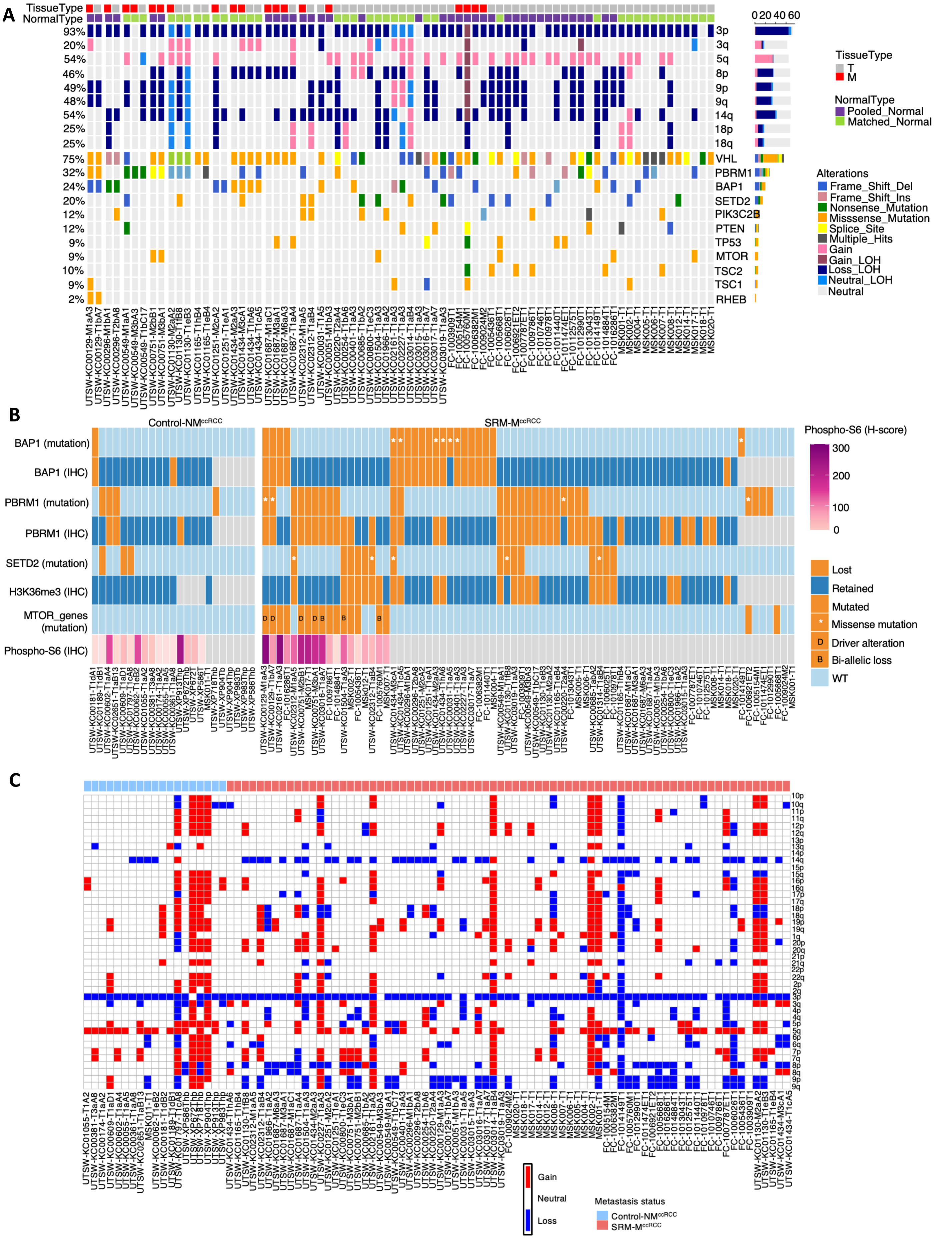

Supplemental Figure 1

D

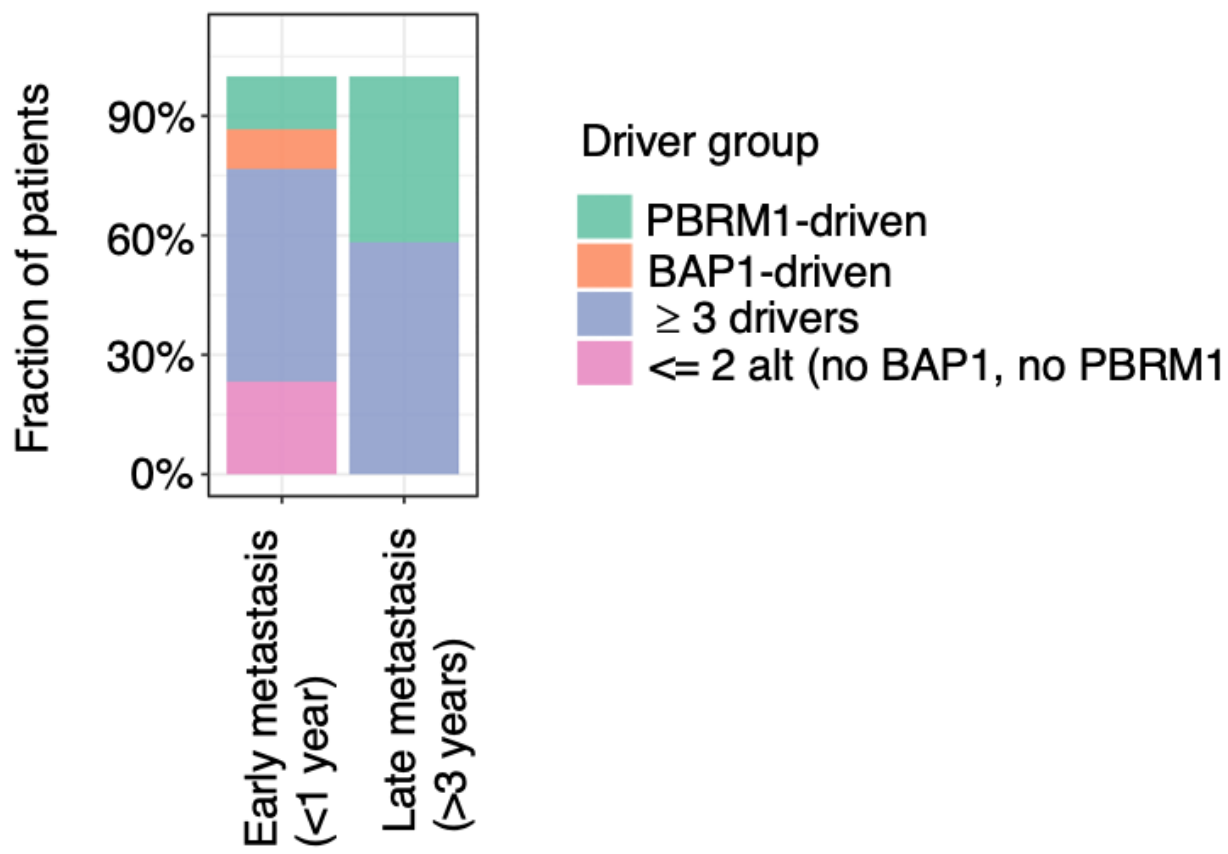

E

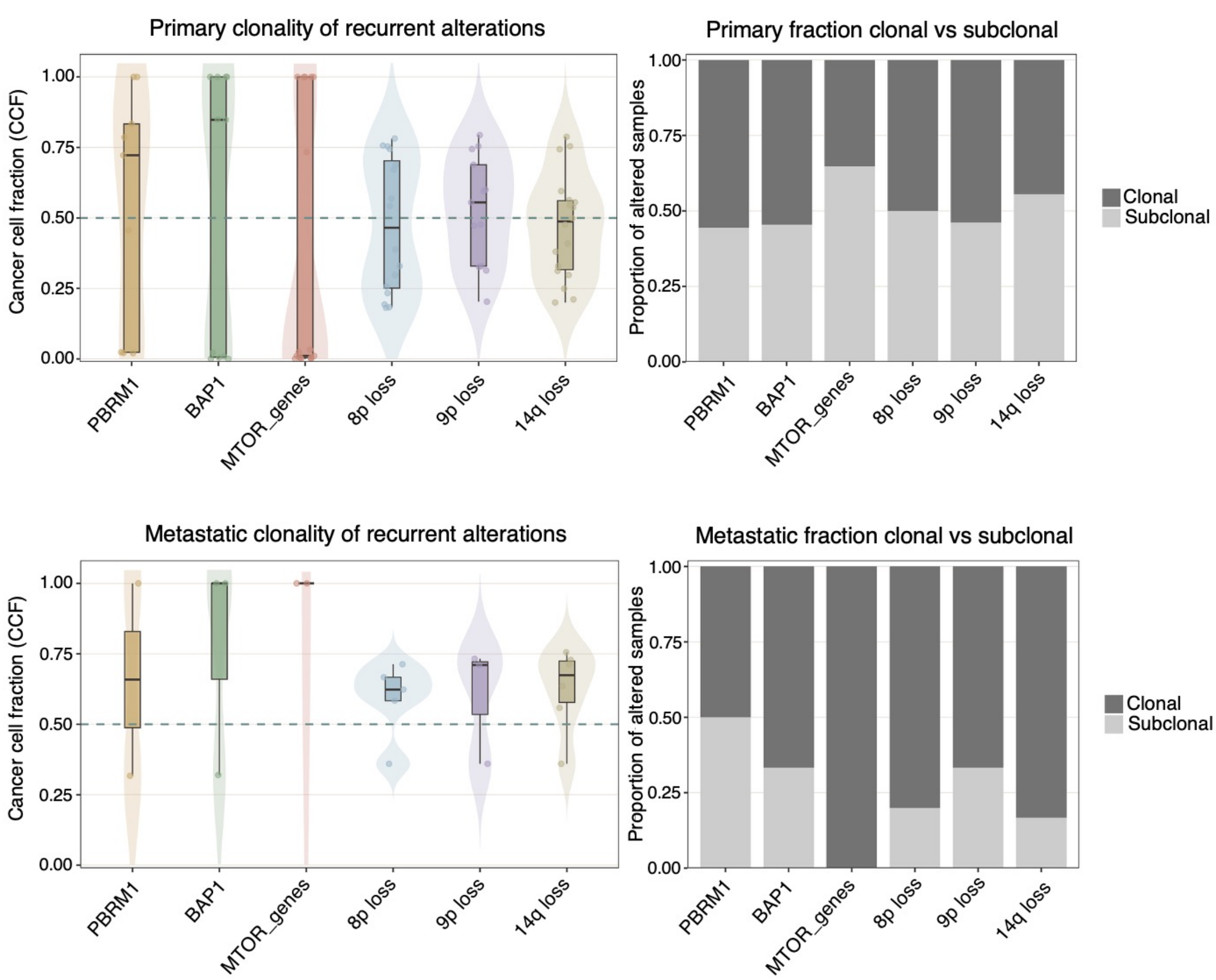

Supplemental Figure 2

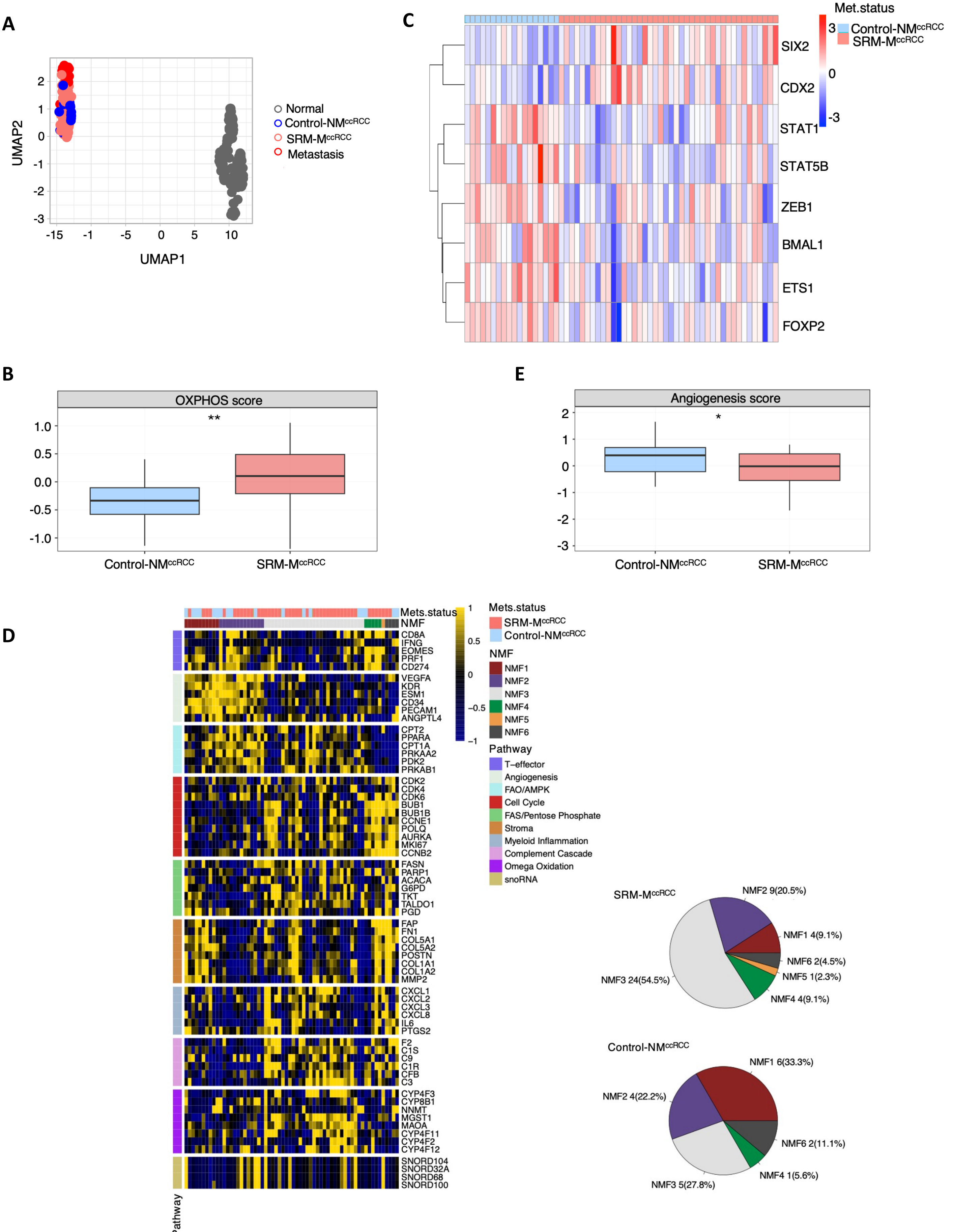

Supplemental Figure 3

A

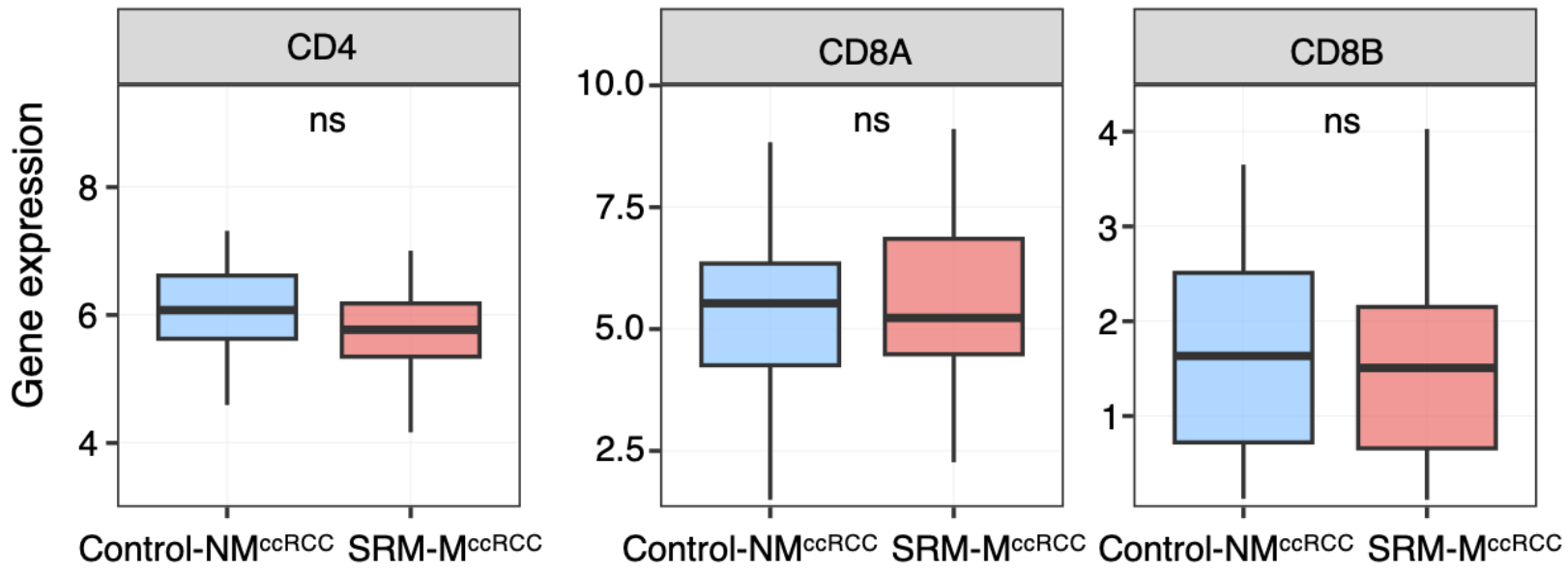

B

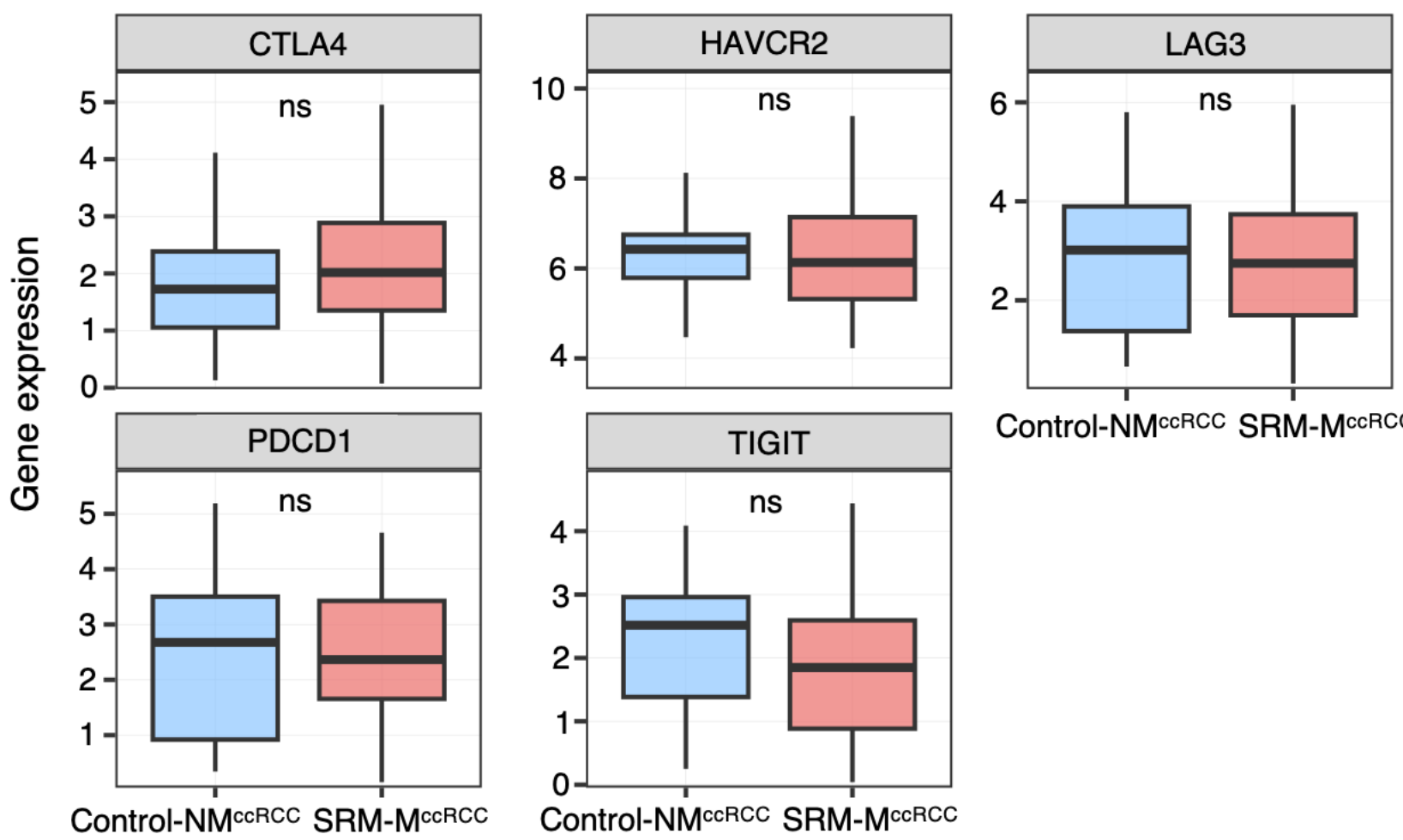

C

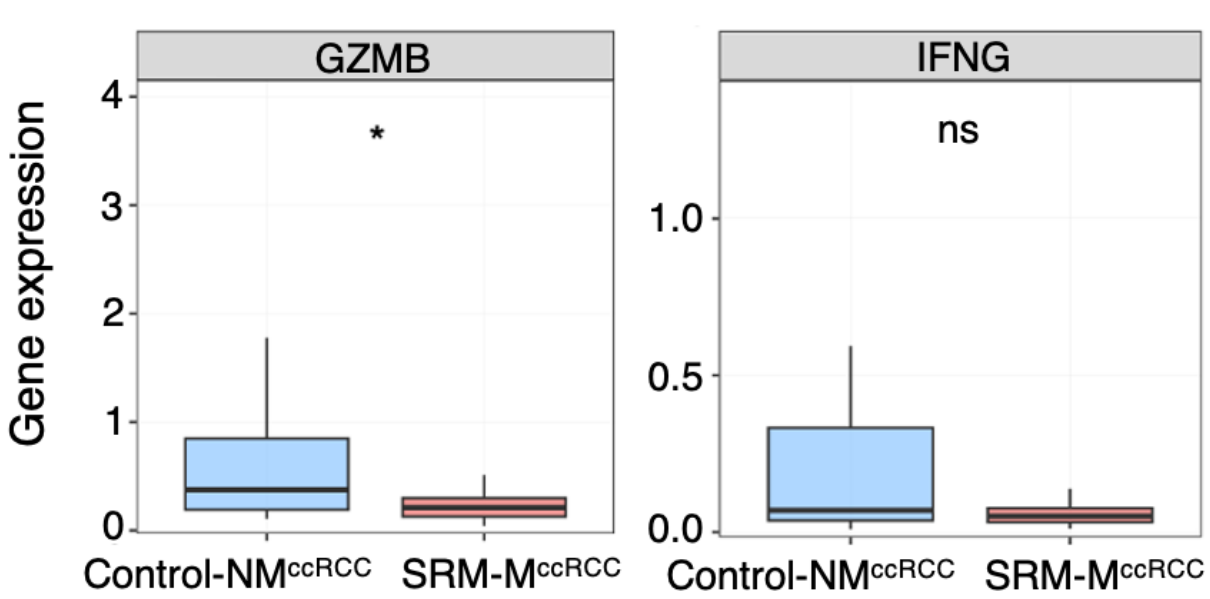

D

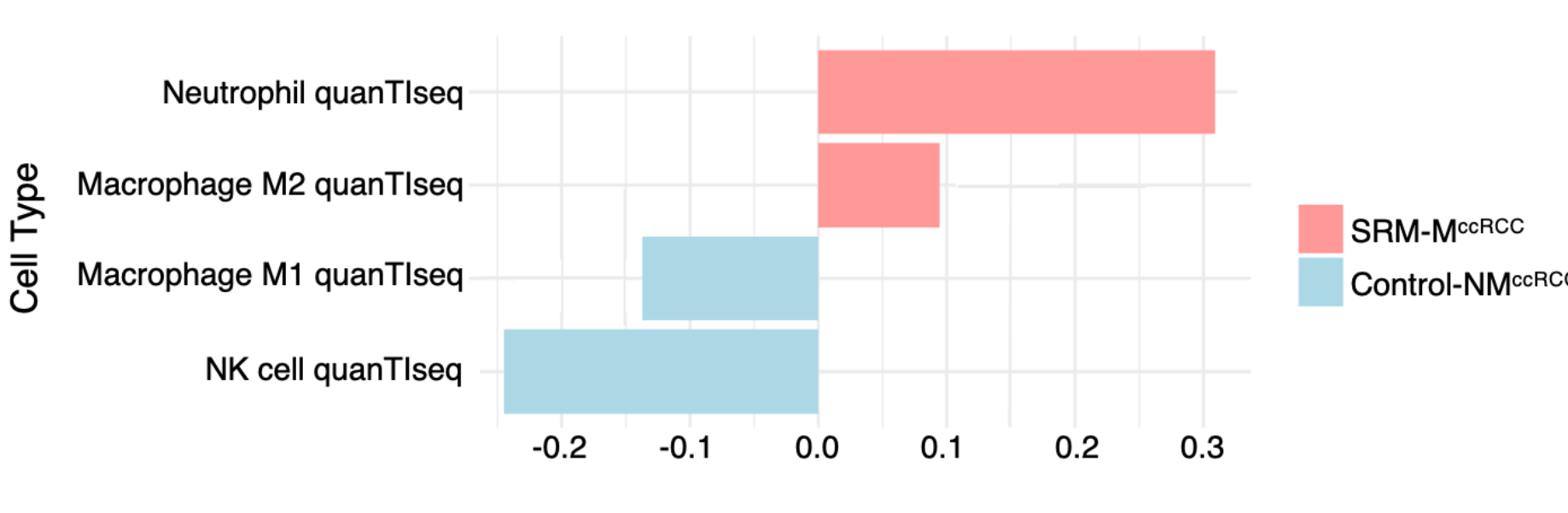

Supplement: Supplementary file 1 [file jciinsight-11-209036-s264.pdf]
